# The RAGE–Ferroptosis Axis Drives Oxidative Stress-Associated Inflammatory Lung Injury in Viral Infection

**DOI:** 10.3390/antiox15040434

**Published:** 2026-03-31

**Authors:** Wenhui Guo, Junhao Luo, Siyu Pu, Simin Cui, Haijun Zhu, Peiqing He, Rongbao Gao

**Affiliations:** 1 NHC Key Laboratory of Biosafety, NHC Key Laboratory of Medical Virology and Viral Diseases, Chinese National Influenza Center, National Institute for Viral Disease Control and Prevention, Chinese Center for Disease Control and Prevention, Beijing 102206, China; guowenhui86@gmail.com (W.G.); luojunhao1998@163.com (J.L.); 17791366202@163.com (S.C.); 18225855528@163.com (H.Z.); becky_hpq@163.com (P.H.); 2 Department of Health Inspection and Quarantine, School of Public Health, Public Health College, Anhui Medical University, Hefei 230032, China

**Keywords:** RAGE, ferroptosis, lung injury, viral infection, oxidative stress, therapeutic target

## Abstract

The receptor for advanced glycation end-products (RAGE) is a lung-enriched pattern recognition receptor implicated in inflammatory responses. Its role in ferroptosis-mediated lung injury during viral infection, however, remains unclear. Here, we combined bioinformatics analysis with in vitro and in vivo experimental validation to investigate the RAGE–ferroptosis axis in influenza virus infection. Cross-analysis of RAGE- and ferroptosis-related genes identified overlapping candidates, suggesting functional crosstalk. Influenza-infected *A549* cells exhibited ferroptotic cell death, characterized by Fe^2+^ accumulation, reactive oxygen species (ROS) elevation, and lipid peroxidation, which was markedly attenuated by the RAGE inhibitor FPS-ZM1. In A/PR/8/34 (H1N1)-infected female *C57BL/6J* mice, FPS-ZM1 treatment improved survival, reduced lung injury, restored redox balance, and modulated key ferroptosis regulators *ACSL4*, *POR*, and *GPX4*. Moreover, RAGE inhibition decreased M1 macrophage and neutrophil infiltration and reduced pro-inflammatory cytokines. Collectively, these findings reveal that RAGE activation drives ferroptosis and amplifies oxidative stress–associated lung injury, whereas RAGE inhibition mitigates tissue damage via the ACSL4/POR/GPX4 pathway and immunomodulation. This study identifies the RAGE–ferroptosis axis as a potential therapeutic target for severe pulmonary inflammation.

## 1. Introduction

Regulated cell death (RCD) plays a central role in the initiation and progression of inflammation-associated tissue injury. Aberrant activation of RCD not only directly leads to the loss of structural cells but also aggravates inflammatory responses and exacerbates tissue damage by triggering innate immune signaling pathways through the release of damage-associated molecular patterns (DAMPs) [1]. In recent years, ferroptosis has emerged as a distinct form of regulated cell death driven by disrupted iron homeostasis and lipid peroxidation. This process is hallmarked by the intracellular accumulation of labile iron, excessive production of reactive oxygen species (ROS), and oxidative damage to polyunsaturated fatty acid–containing phospholipids, ultimately culminating in loss of membrane integrity and cell death [2,3]. Accumulating evidence indicates that ferroptosis has been extensively implicated in the pathogenesis of diverse conditions, including acute lung injury (ALI), acute respiratory distress syndrome (ARDS), and cardiovascular diseases. It is increasingly recognized as a crucial molecular nexus linking oxidative stress, inflammatory responses, and tissue damage and represents a potential target for therapeutic intervention [4,5,6].

In the inflammatory microenvironment, ferroptosis is not an isolated event; rather, its activation is orchestrated by multiple upstream signals and intricately interlinked with inflammatory pathways, creating self-amplifying feedback loops that exacerbate tissue injury [7]. Lipid peroxidation products and ROS generated during ferroptosis not only directly compromise cellular integrity but also act as amplifiers of inflammatory signaling, promoting sustained expression of pro-inflammatory cytokines such as interleukin-6 (IL-6) and tumor necrosis factor-alpha (TNF-α), as well as the recruitment of immune cells [8]. In addition, pattern-recognition receptors (PRRs), including Toll-like receptors (TLRs) and retinoic acid-inducible gene I–like receptors (RLRs), have been reported to sense DAMPs and trigger oxidative stress responses, thereby establishing cross-regulatory interactions with the ferroptosis process [9]. However, whether and how ferroptosis is finely regulated within PRR-mediated inflammatory signaling networks remains to be fully elucidated at the molecular level.

The influenza virus, a highly variable respiratory pathogen, can induce acute inflammatory lung injury [10,11]. Previous studies have shown that influenza virus infection perturbs cellular iron homeostasis and antioxidant defense systems, particularly the cystine/glutamate antiporter (system Xc^−^)/glutathione (GSH)/glutathione peroxidase 4 (GPX4) axis, leading to increased iron accumulation and lipid peroxidation, thereby triggering ferroptosis and exacerbating lung tissue damage [12,13,14,15]. Additionally, targeting ferroptosis has shown therapeutic potential to simultaneously inhibit viral replication and mitigate tissue injury, including interventions with proanthocyanidins and indoleamine 2,3-dioxygenase 1 [14,15,16]. The receptor for advanced glycation end products (RAGE) is a key PRR that senses endogenous DAMPs, including high mobility group box 1 (HMGB1) and S100 proteins, and activates downstream signaling pathways such as nuclear factor kappa B (NF-κB) and activator protein 1 (AP-1) to regulate inflammatory responses and oxidative stress. In addition, RAGE contributes to tissue injury by modulating autophagy and multiple forms of cell death [17]. Our previous study and others have demonstrated that RAGE contributes to inflammation-induced injury in severe influenza, including cytokine induction, and can serve as a therapeutic target to mitigate pathogenicity and mortality [18,19]. These findings are consistent with earlier reports showing that RAGE deficiency improves survival and reduces lung damage during influenza A virus pneumonia [20]. Moreover, recent evidence further indicates that the RAGE signaling axis mediates ferroptosis in cardiomyocytes in a heart failure model, suggesting a direct role in the regulation of ferroptosis [21].

However, the molecular mechanisms by which RAGE engages the ferroptosis signaling axis in inflammation-associated lung injury remain to be elucidated. Therefore, in the present study, we employed *influenza A virus* (*IAV*) infection as in vitro and in vivo models of inflammatory stimulation to systematically investigate the functional interplay between RAGE and ferroptosis. Through integrative analysis of public databases, in vitro infection models, and mouse experiments, we demonstrate that RAGE activation promotes key components of the ferroptotic machinery and amplifies lipid peroxidation and inflammatory responses via the acyl-CoA synthetase long-chain family member 4 (ACSL4)/cytochrome P450 oxidoreductase (POR)/GPX4 axis, further driving pro-inflammatory macrophage polarization and lung tissue damage. Both in vitro and in vivo administration of the RAGE inhibitor FPS-ZM1 effectively attenuates ferroptosis and mitigates lung injury. These findings unveil the central role of the RAGE–ferroptosis signaling axis in inflammatory lung injury and provide novel mechanistic insights for intervention strategies targeting host-mediated damage pathways.

## 2. Materials and Methods

### 2.1. Bioinformatic Analysis of RAGE and Ferroptosis Pathways

Human gene databases (GeneCards, https://www.genecards.org/) were searched using the keywords “RAGE,” “Ferroptosis,” and “influenza virus infection” to retrieve RAGE-, ferroptosis-, and influenza virus-related genes. The identified RAGE and ferroptosis genes were input into the STRING database (version 11.0; https://string-db.org/(accessed on 15 March 2025)) to construct a protein–protein interaction (PPI) network with a minimum required interaction score of 0.4 (medium confidence), aiming to explore potential interactions between RAGE and downstream ferroptosis signaling. The PPI network was visualized and analyzed using Cytoscape software(version 3.10.3), and the top 30 hub genes ranked by degree were selected for further analysis. The overlapping genes between RAGE and ferroptosis were cross-analyzed with influenza virus-related genes to assess potential involvement of the shared signaling network in viral infection.

Venn analysis of RAGE- and ferroptosis-related genes, as well as the shared RAGE–ferroptosis genes with influenza virus-related genes, was performed using R software (version 4.3.2). Gene Ontology (GO) analysis and Kyoto Encyclopedia of Genes and Genomes (KEGG) pathway enrichment analysis were conducted on the overlapping RAGE–ferroptosis genes. GO analysis was employed to categorize gene functions and describe gene product roles in biological processes, cellular components, and molecular functions. KEGG pathway analysis was performed to identify potential signaling pathways, and pathways with adjusted *p* < 0.05 and containing at least five genes were considered significantly enriched.

### 2.2. Cell Culture and Treatments

*A549* and *THP-1* cells were obtained from the Influenza Laboratory of the Chinese Center for Disease Control and Prevention and maintained in Dulbecco’s Modified Eagle Medium (DMEM, Gibco, Waltham, MA, USA, Cat# 11965092) supplemented with 10% fetal bovine serum (FBS, Gibco, USA, Cat# 26010074) and 1% penicillin–streptomycin (Gibco, USA, Cat# 15240096) at 37 °C with 5% CO_2_.

For infection experiments, *A549* cells were seeded in 96-well plates (3 × 10^4^ cells per well) or 6-well plates (1.5 × 10^5^ cells per well) and grown to 70–90% confluence. A549 cells were infected with *Influenza A virus* A/California/04/2009 (H1N1, CA04), a human isolate strain that replicates efficiently in human epithelial cells and was used for all in vitro experiments, at a multiplicity of infection (MOI) of 1.0 in infection medium consisting of DMEM supplemented with 0.2% bovine serum albumin (BSA, Sigma-Aldrich, St. Louis, MO, USA, Cat# A1933), 2.5% 4-(2-hydroxyethyl)-1-piperazineethanesulfonic acid (HEPES, Gibco, USA, Cat# 15630106), 1% penicillin–streptomycin (Gibco, USA, Cat# 15240096), and 2 µg/mL Trypsin treated with L-(tosylamido-2-Phenyl)ethyl Chloromethyl Ketone(TPCK)-treated trypsin (Thermo Scientific, Waltham, MA, USA, Cat# 20233). After 1 h of incubation at 35 °C, the viral inoculum was removed, and cells were washed three times with phosphate-buffered saline (PBS, Solarbio, Beijing, China, Cat# P1020). Cells were then incubated in fresh infection medium with the same composition as described above with or without inhibitors: the FPS-ZM1 group received 8 µM FPS-ZM1 (Selleck, Houston, TX, USA, Cat# S8185), the Fer-1 group received 3.125 µM Ferrostatin-1 (MCE, Beijing, China, Cat# HY-100579), and the CA04 group received infection medium alone (vehicle control). FPS-ZM1 and Ferrostatin-1 were dissolved in dimethyl sulfoxide (DMSO) to prepare 10 mM stock solutions for their respective working concentrations. The final DMSO concentration (≤0.1%) was kept identical across all experimental groups. The working concentration of FPS-ZM1 was based on previous studies, whereas the optimal concentration of Fer-1 was determined as 3.125 µM via dose–response experiments (Figure S1A).

Simultaneously with *A549* infection, *THP-1* cells seeded in 96-well plates (3 × 10^4^ cells per well) were differentiated into monocyte-derived macrophages by treatment with 185 ng/mL phorbol-12-myristate-13-acetate (PMA, Solarbio, China, Cat# P6741, dissolved in DMEM) for 24 h. At 24 h post-infection, *A549* cells were harvested for mRNA extraction, and culture supernatants were collected and transferred onto differentiated *THP-1* cultures. *THP-1* cells were not directly treated with inhibitors; instead, they were exposed to *A549*-derived supernatants. Prior to supernatant transfer, *THP-1* culture medium was aspirated, and cells were gently washed three times with PBS. After 48 h of incubation, differentiated *THP-1* cells were subsequently analyzed. The non-toxic concentrations of FPS-ZM1 (8 μM) and Fer-1 (3.125 μM) in *THP-1* cells were also confirmed to be non-cytotoxic (Figure S1B,C). All procedures were conducted in a biosafety level 2 (BSL-2) laboratory.

### 2.3. Cell Counting Kit-8 (CCK-8) Assay

Cell viability was assessed using the CCK-8 assay (G-CLONE, Tianjin, China, Cat# GK10001). Cells were seeded in 96-well plates (3 × 10^4^ cells per well), with four technical replicates per condition. Prior to detection, the culture medium was aspirated, and 100 µL of CCK-8 working solution diluted 1:10 in culture medium was added to each well. After incubation at 37 °C for 30 min, the medium was gently mixed, and absorbance at 450 nm was measured using a microplate reader (Thermo Fisher Scientific, USA). Cell viability was calculated as the ratio of the optical density (OD) of treated wells to that of blank wells.

### 2.4. Iron and SOD Measurement

Iron levels in cells and mouse lung tissues, as well as lung superoxide dismutase (SOD) activity, were measured using commercial kits. Cells were seeded in 6-well plates (1.5 × 10^5^ cells per well), with three technical replicates per condition. After removal of excess fluid, lung tissues were homogenized and sonicated in ice-cold lysis buffer. Sonication parameters were set at 150 W, 3 s on and 7 s off, for a total of 3 min. SOD (Nanjing Jiancheng Bioengineering Institute, Nanjing, China, Cat# A001-3), Fe^3+^ (Applygen, Shanghai, China, Cat# E1041), and Fe^2+^ (Applygen, China, Cat# E1042) were quantified according to the manufacturers’ instructions.

### 2.5. RNA Extraction and Quantitative Real-Time PCR (qRT-PCR)

Total RNA was extracted from *A549* cells seeded in 96-well plates (3 × 10^4^ cells per well) using a cell/tissue total RNA extraction kit (TIANGEN, Beijing, China, Cat# DP430) according to the manufacturer’s instructions. Four technical replicates were included per condition. To quantify the relative expression levels of *ACSL4*, *POR*, and *GPX4*, qRT-PCR was performed by the BeyoFast™ SYBR Green One-Step qRT-PCR Kit (Beyotime, Beijing, China, Cat# D7260) on a real-time PCR detection system (Agilent Technologies Inc., Santa Clara, CA, USA). The housekeeping gene *glyceraldehyde-3-phosphate dehydrogenase (GAPDH)* was used as the internal control. The accession numbers of the analyzed genes were as follows: *ACSL4* (NM_022977.4), *POR* (NM_000941.3), *GPX4* (NM_002085.5), and *GAPDH* (NM_002046.7). The specific primer sets were used as follows: *ACSL4* forward: GCTATCTCCTCAGACACACCGA, *ACSL4* reverse: AGGTGCTCCAACTCTGCCAGTA; *POR* forward: ACTCTGCTCTCGTCAACCAGCT, *POR* reverse: TGGGTGCTTCTTGTTGGACTCC; *GPX4* forward: ACAAGAACGGCTGCGTGGTGAA, *GPX4* reverse: GCCACACACTTGTGGAGCTAGA; *GAPDH* forward: GTCTCCTCTGACTTCAACAGCG; *GAPDH* reverse: ACCACCCTGTTGCTGTAGCCAA. Relative gene expression levels were calculated using the 2^(−ΔΔCt)^ method.

### 2.6. Immunofluorescence and High-Content Imaging

Intracellular ROS, lipid peroxidation, and *THP-1* macrophage polarization were assessed by immunofluorescence using a high-content imaging system. Prior to ROS and lipid peroxidation detection, culture supernatants of *A549* and *THP-1* cells in 96-well plates (3 × 10^4^ cells per well) were aspirated, and cells were gently washed once with PBS. Cells were subsequently processed using a ROS Assay Kit (Beyotime, China, Cat# S0033M) or BODIPY 581/591 C11 (Beyotime, China, Cat# S0043M) according to the manufacturer’s instructions. For lipid peroxidation analysis, oxidized lipids were detected as green fluorescence, whereas non-oxidized (reduced) lipids were detected as red fluorescence.

For polarization analysis, *THP-1* cells were washed with PBS and incubated with anti-CD86 (1:500; MCE, China, Cat# HY-P80609) and anti-CD206 (1:500; Proteintech, Wuhan, China, Cat# 60143-1-Ig) antibodies at 37 °C for 1 h, followed by three PBS washes. Alexa Fluor 488-conjugated secondary antibody-conjugated (1:500; Lablead, Beijing, China, Cat# Y1048) and Alexa Fluor 594-conjugated (1:500; Lablead, China, Cat# Y1106) secondary antibodies were used to label CD86 and CD206, respectively, with 1 h incubation at 37 °C and subsequent PBS washes. Nuclei were counterstained with 80 µL of Hoechst 33342 (Beyotime, China, Cat# C1022) per well at 37 °C for 15 min, followed by three PBS washes. Finally, 100 µL PBS was added to each well, and images were captured using a high-content imaging system (Moldev, Tokyo, Japan) and analyzed with ImageJ software (version 1.53a, NIH, Bethesda, MD, USA).

For quantitative analysis, four randomly selected fields per well were captured under identical acquisition parameters. The mean fluorescence intensity (MFI) of the corresponding fluorescence signals was calculated for each field using ImageJ software. The averaged MFI per well was treated as one technical replicate, and four technical replicates were included per condition, resulting in a total of 16 images analyzed per group.

### 2.7. Animal Experimentation

All animal experiments were conducted in accordance with the guidelines approved by the Institutional Animal Care and Use Committee of the National Institute for Viral Disease Control and Prevention, China CDC (ethical approval number: 20221107112; approval date: 1 January 2023). Female *C57BL/6J* mice (8–10 weeks old) were randomly allocated to experimental groups prior to infection, housed under specific pathogen-free conditions, with five mice per cage, and maintained on a 12 h light/dark cycle with free access to food and water. Mice were anesthetized with isoflurane (provided by the animal facility) and intranasally (i.n.) inoculated with 2.5 × 10^2^ 50% tissue culture infectious dose (TCID_50_) (10 × LD_50_) of A/PR/8/34 (H1N1, PR8) virus, a well-characterized mouse-adapted strain that induces robust and reproducible lung pathology in C57BL/6J mice, diluted in 20 µL PBS (Solarbio, China, Cat# P1020). Mice receiving PBS alone served as MOCK controls (*n* = 20). FPS-ZM1 was dissolved and diluted according to the manufacturer’s instructions in a vehicle solution containing 10% DMSO (Solarbio, China, Cat# D8370), 40% PEG300 (Solarbio, China, Cat# P8430), 5% Tween-80 (Solarbio, China, Cat# T8360), and 45% PBS. From days 2 to 5 post-infection, mice in the FPS-ZM1 group (*n* = 20) received intraperitoneal injections of FPS-ZM1 at 2 mg/kg every 24 h, the PR8 group (*n* = 20) and the Mock group received an equal volume of the same vehicle solution (10% DMSO, 40% PEG300, 5% Tween-80, and 45% PBS) without FPS-ZM1 on the same schedule. Body weight (*n* = 10 per group) was monitored daily for up to 14 days or until death. Mice that lost more than 25% of their initial body weight were humanely euthanized. No other animals or data points were excluded. On days 4 and 8 post-infection, five mice per group were randomly selected and euthanized for the collection of bronchoalveolar lavage fluid (BALF) and lung tissues for downstream analyses.

### 2.8. Hematoxylin and Eosin (H&E) and Immunohistochemistry (IHC)

Hematoxylin and eosin (H&E) staining was performed for histopathological evaluation. Lung tissue samples were fixed in 4% neutral formalin (Solarbio, China, Cat# D8370) for 24 h, dehydrated through a graded ethanol series, and embedded in paraffin. Tissue sections were cut at 5 µm thickness. H&E staining was performed using an H&E Stain Kit (Solarbio, China, Cat# G1120) following standard protocols, and the severity of lung pathology was scored accordingly. Specifically, the slides were randomized, read, and subsequently scored using a semi-quantitative 0–4 scale assessing alveolar edema, inflammatory infiltration, hemorrhage, and tissue necrosis, as previously described. 0 = no injury, 1 = mild, 2 = moderate, 3 = severe, and 4 = very severe [22].

For immunohistochemistry (IHC), tissue sections were deparaffinized in xylene (Solarbio, China, Cat# IX0270) and rehydrated through a graded ethanol series. Antigen retrieval was performed in citrate buffer (Solarbio, China, Cat# C1010, pH 6.0) at 95 °C for 15 min. Endogenous peroxidase activity was blocked with the peroxidase blocking reagent provided in the Two-Step Immunohistochemical Detection Kit (ZSGB-BIO, Beijing, China, Cat# PV-9001) for 10 min at room temperature, and non-specific binding was minimized by incubating sections in 5% normal goat serum (Solarbio, China, Cat# SL038) for 30 min at room temperature. Sections were then incubated with a primary antibody against 4-hydroxynonenal (4-HNE, 1:2000, MCE, China, Cat# HY-P81208) overnight at 4 °C. After washing with PBS, sections were sequentially incubated with a reaction enhancer and goat anti-rabbit secondary antibody (provided in the PV-9001 kit) for 1 h at room temperature. 3,3′-diaminobenzidine (DAB, ZSGB-BIO, China, Cat# ZLI-9019) was used for color development, and nuclei were counterstained with hematoxylin. Sections were dehydrated, cleared, and mounted with neutral resin (Solarbio, China, Cat# G8590).

Images were captured using a Zeiss microscope under identical exposure settings. For each lung section, five randomly selected high-power fields were acquired. Five mice per group were analyzed at each time point (day 4 and day 8 post-infection), resulting in a total of 25 images per group per time point. Quantification was performed using ImageJ software by calculating the percentage of positive staining area relative to the total tissue area. The averaged value per mouse was used for statistical analysis.

### 2.9. Western Blotting

*A549* cells were infected with CA04 (MOI = 1) for 24 h, followed by treatment with PBS, 8 μM FPS-ZM1 or 3.125 μM Ferrostatin-1 (Fer-1). Cells were collected and lysed in radioimmunoprecipitation assay (RIPA) buffer (Solarbio, China, Cat# R0010) supplemented with 1% protease and phosphatase inhibitors (Yuanye Bio-Technology, Shanghai, China, Cat# R32811). Mouse lung tissues were homogenized in RIPA lysis buffer (Solarbio, China, Cat# R0010) supplemented with 1% protease and phosphatase inhibitors (Yuanye Bio-Technology, China, Cat# R32811) using a tissue homogenizer. Homogenates were centrifuged, and the supernatants were mixed with loading buffer (provided with precast gels) and boiled at 100 °C for 10 min. Protein samples were separated on SDS–polyacrylamide gels (Lablead, China, Cat# P42015) and transferred onto 0.45 μm polyvinylidene fluoride (PVDF) membranes (Biosharp, Beijing, China, Cat# BS-PVDF-45). Membranes were blocked with 5% BSA (Sigma-Aldrich, USA, Cat# A1933) at room temperature for 2 h, followed by overnight incubation at 4 °C with primary antibodies against GPX4 (1:1000, RayBiotech, Peachtree Corners, GA, USA, Cat# 144-01933), POR (1:1000, RayBiotech, USA, Cat# 144-08142), ACSL4 (1:1000, RayBiotech, USA, Cat# 144-06826), STAT3 (1:2000, Abcam, Waltham, MA, USA, Cat# ab31370), TNF-α (1:500, Abcam, USA, Cat# ab215188), or β-Actin (1:2000, Bioss, Beijing, China, Cat# bsm-63325R) as loading control. After washing with Tris-buffered saline with Tween-20 (TBST, Solarbio, China, Cat# T1085), membranes were incubated with horseradish peroxidase (HRP)-conjugated secondary antibody (1:5000, Abcam, Cambridge, UK, Cat# ab6721) for 1 h at room temperature. Protein signals were visualized using SuperSignal West Pico PLUS Chemiluminescent Substrate (Thermo Fisher Scientific, USA, Cat# 34579) and imaged with a chemiluminescence imaging system (Bio-Rad, Hercules, CA, USA). Band intensities were quantified using ImageJ software and normalized to β-Actin (Figure S2).

### 2.10. Enzyme-Linked Immunosorbent Assay (ELISA)

The concentration of IL-6 in mouse sera and lung tissues was determined using an ELISA kit (Elabscience, Wuhan, China, Cat# E-EL-M0044) according to the manufacturer’s instructions. Serum samples were diluted 1:10 in PBS, and lung tissue homogenates were diluted 1:20 in PBS. All assays were performed according to the kit’s protocols.

### 2.11. Flow Cytometry

Bronchoalveolar lavage fluid (BALF) cells were collected from mice on day 8 post-PR8 infection. Briefly, the lungs were lavaged three times with 500 μL pre-cooled PBS (Solarbio, China, Cat# P1020), and the recovered fluid (~1.5 mL) was pooled in a centrifuge tube. Cells were centrifuged at 300× *g* for 5 min at 4 °C, and the supernatant was discarded. Red blood cells were lysed using red blood cell (RBC) lysis buffer (Solarbio, China, Cat# R1010) at room temperature for 10 min, followed by washing with an equal volume of cell staining buffer (Elabscience, China, Cat# E-CK-A107) and centrifugation. Cells were resuspended in cell staining buffer and stained with a fixable viability dye according to the manufacturer’s instructions (Invitrogen, Carlsbad, CA, USA, Cat# E1169) at 4 °C for 15 min in the dark to exclude dead cells. After washing, cells were incubated with anti-mouse Fc receptor blocking antibody (Elabscience, China, Cat# E-AB-F0997A) at 4 °C for 10 min to minimize non-specific binding. Cells were then stained with fluorophore-conjugated antibodies: CD45-Violet 450 (Elabscience, China, Cat# E-AB-F1136Q), CD11b-FITC (Elabscience, China, Cat# E-AB-F1081C), F4/80-PE (Elabscience, China, Cat# E-AB-F0995D), CD86-APC (Elabscience, China, Cat# E-AB-F0994E), CD206-PE/Cy7 (Elabscience, China, Cat# E-AB-F1135H), and Ly6G-APC (Elabscience, China, Cat# E-AB-F1108E) at 4 °C for 30 min in the dark. After three washes with cell staining buffer, cells were resuspended in 200 μL staining buffer and analyzed on a flow cytometer (BD, Franklin Lakes, NJ, USA). Data were analyzed using FlowJo software (version 10.8.1; BD Biosciences, Franklin Lakes, NJ, USA). Macrophage subsets were defined as M1 (CD45^+^CD11b^+^F4/80^+^CD86^+^), M2 (CD45^+^CD11b^+^F4/80^+^CD206^+^), and neutrophils were defined as CD45^+^CD11b^+^Ly6G^+^ (Figure S3).

### 2.12. Statistical Analysis

Mouse survival curves were analyzed using the Kaplan–Meier method and compared using the log-rank (Mantel–Cox) test. For two-group comparisons, an unpaired two-tailed Student’s *t*-test was used. For in vitro experiments, each treatment group was compared separately with the CA04 group; for in vivo experiments, treated groups were compared separately with the PR8 group. Correlation analyses were performed using Pearson’s or Spearman’s correlation coefficients, as appropriate. Western blot densitometry was conducted by normalizing protein expression to β-actin, and results were visualized using R. A two-tailed *p*-value < 0.05 was considered statistically significant. All statistical analyses were performed using GraphPad Prism (version 10.0; GraphPad Software, San Diego, CA, USA). The Graphic Abstract was created with BioRender.com.

## 3. Results

### 3.1. Cross-Analysis of RAGE and Ferroptosis Effector Molecular Networks

**Figure 1:**
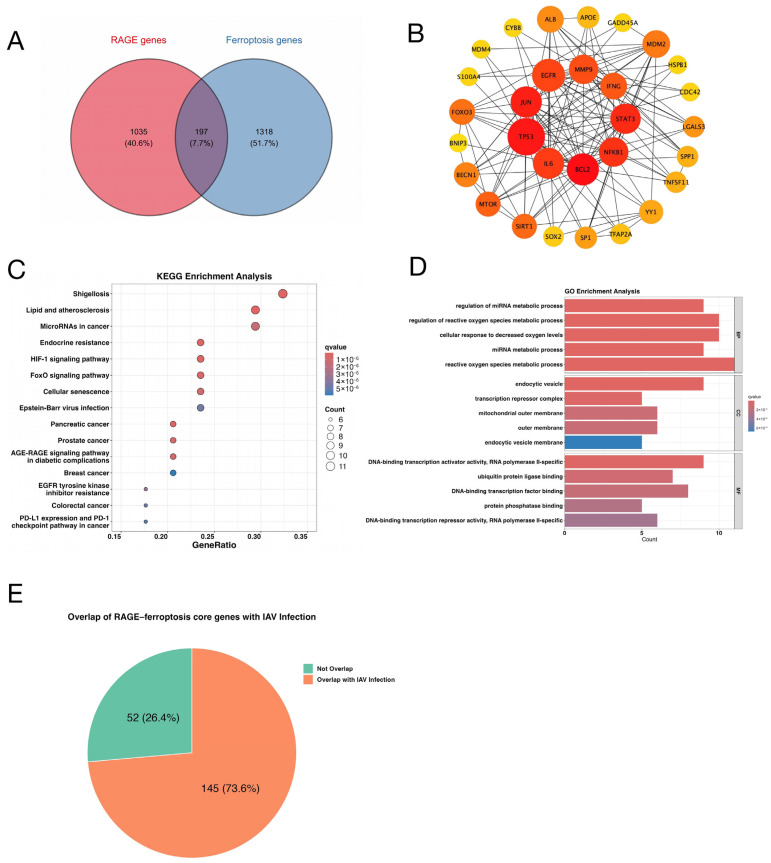
Shared Molecular Landscape of RAGE and Ferroptosis. (**A**) Venn diagram showing 197 overlapping genes between receptor for advanced glycation end-products (RAGE, pink) and ferroptosis-related genes (blue), identified as core candidates for subsequent mechanistic analysis. (**B**) Protein–protein interaction (PPI) network. Nodes represent proteins, and edges indicate experimentally validated or predicted interactions. Node size and color intensity correspond to combined confidence scores from the STRING database. (**C**) KEGG pathway enrichment analysis of the overlapping genes. (**D**) Gene Ontology (GO) biological process (BP) enrichment analysis. Colors indicate the q-value of enrichment, and the x-axis represents the number of enriched genes. (**E**) Distribution of RAGE–ferroptosis overlapping genes annotated as associated with influenza virus infection.

Although previous studies have suggested a role for RAGE-mediated inflammatory signaling and ferroptosis in infection-induced lung injury, the molecular mechanisms underlying their potential crosstalk remain unclear. To systematically evaluate the interaction between the RAGE pathway and ferroptosis, we retrieved 1232 RAGE-related genes and 1515 ferroptosis-related genes from the GeneCards database. Cross-comparison identified 197 overlapping genes, representing candidate genes potentially mediating crosstalk between the two pathways (Figure 1A).

To further elucidate the interactions among the overlapping genes and their functional roles within the signaling network, a protein–protein interaction (PPI) network was constructed and visualized using Cytoscape, followed by topological analysis. The top 30 nodes ranked by degree were identified as core nodes. The network comprised 140 edges, with an average node degree of 9.33 (Figure 1B). Key genes, including *TP53*, *EGFR*, *BCL2*, *JUN*, *STAT3*, and *IL6*, interacted with more than 15 nodes, suggesting that they may serve as hub genes mediating the functional coupling between RAGE signaling and ferroptosis-related pathways.

Subsequently, KEGG pathway enrichment analysis of the overlapping genes was performed, revealing significant enrichment in pathways associated with inflammatory responses, oxidative stress, and regulation of cell fate (Figure 1C, Table 1). These pathways included Shigellosis, Endocrine resistance, Lipid and atherosclerosis, HIF-1 signaling pathway, Pancreatic cancer, FoxO signaling pathway, Prostate cancer, AGE-RAGE signaling pathway in diabetic complications, Cellular senescence, MicroRNAs in cancer, EGFR tyrosine kinase inhibitor resistance, Epstein–Barr virus infection, Colorectal cancer, and PD-L1 expression and PD-1 checkpoint pathway in cancer. The established roles of these pathways in infection, immune regulation, and stress responses, suggest potential functional convergence through shared inflammatory and metabolic pathways.

GO analysis was conducted using the clusterProfiler R package(version 4.8.1; https://bioconductor.org/packages/clusterProfiler/, accessed on 15 March 2025). The results identified significantly enriched biological terms across biological process (BP), cellular component (CC), and molecular function (MF) categories (adjust *p*-value < 0.05). The top 10 enriched terms in each category were visualized as bar plots (Figure 1D, Table 2). In the BP category, the top enriched terms primarily included regulation of miRNA metabolic process, regulation of reactive oxygen species metabolic process, cellular response to decreased oxygen levels, miRNA metabolic process, and reactive oxygen species metabolic process. In the MF category, the top enriched terms primarily included DNA-binding transcription activator activity, RNA polymerase II-specific, ubiquitin protein ligase binding, DNA-binding transcription factor binding, and protein phosphatase binding. For the CC category, the most enriched terms were endocytic vesicle, transcription repressor complex, mitochondrial outer membrane, outer membrane, and endocytic vesicle membrane. Further analysis revealed that 73.6% of the key molecules mediating the interaction between ferroptosis and RAGE were activated following influenza virus infection (Figure 1E). Therefore, the influenza infection model was selected for subsequent investigations.

### 3.2. RAGE Inhibitor Attenuates CA04-Induced Injury in A549 Cells by Suppressing Ferroptosis

**Figure 2:**
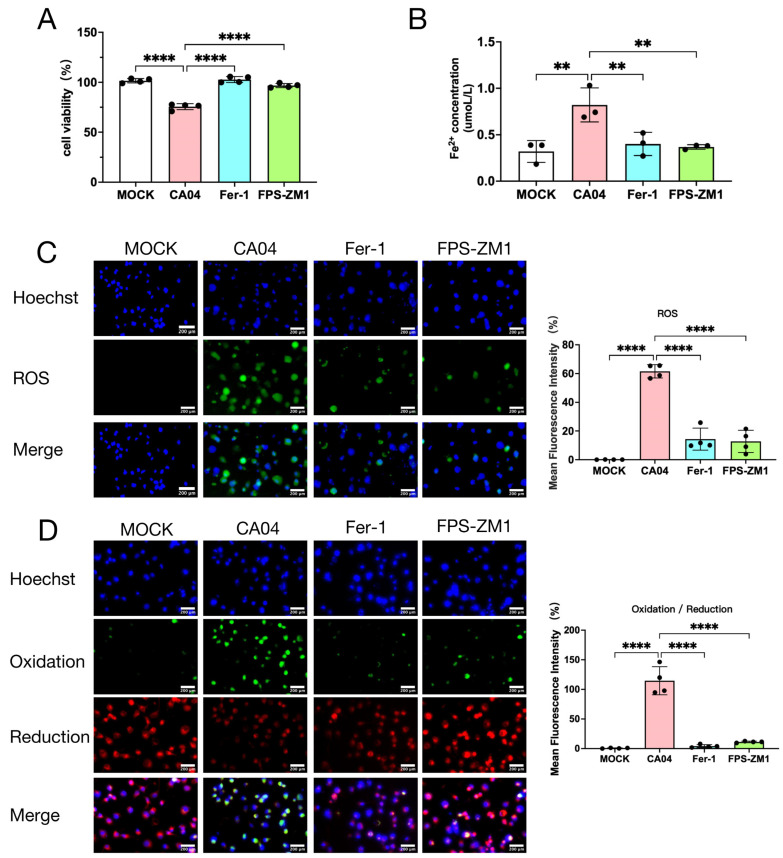
Inhibition of RAGE reduces ferroptosis levels following CA04 infection. (**A**) Cell viability of CA04-infected A549 cells after 24 h treatment with Fer-1 or FPS-ZM1 (*n* = 4). (**B**) Intracellular Fe^2+^ levels in CA04-infected A549 cells after 24 h treatment with Fer-1 or FPS-ZM1 (*n* = 3). (**C**) Immunofluorescence detection of reactive oxygen species (ROS, green) and nuclei (DAPI, blue) in CA04-infected A549 cells after 24 h treatment with Fer-1 or FPS-ZM1 (*n* = 4). Scale bar = 200 μm. (**D**) Immunofluorescence detection of lipid peroxidation in CA04-infected A549 cells after 24 h treatment with Fer-1 or FPS-ZM1. Representative images show oxidized lipids (green), reduced lipids (red), and nuclei (DAPI, blue). Scale bar = 200 μm. Quantification represents the ratio of Mean Fluorescence Intensity (MFI) of oxidized to reduced BODIPY 581/591 C11 (*n* = 4). Data are presented as mean ± standard deviation (SD). Statistical comparisons were performed between each group and the CA04 group. ** *p* < 0.01, **** *p* < 0.0001.

To investigate whether RAGE mediates influenza virus-induced cellular injury through the regulation of ferroptosis, A549 cells were infected with CA04 and treated with the RAGE inhibitor FPS-ZM1 or ferroptosis inhibitor Ferrostatin-1 (Fer-1). Cell viability assays showed that both Fer-1 and FPS-ZM1 treatment significantly increased A549 cell survival compared with the CA04 group at 24 h post-infection (Figure 2A). Measurement of intracellular Fe^2+^ revealed that CA04 infection markedly elevated Fe^2+^ levels, while both Fer-1 and FPS-ZM1 treatment significantly reduced Fe^2+^ accumulation (Figure 2B). ROS levels detected using a fluorescent probe demonstrated that CA04 infection induced substantial ROS accumulation, which was markedly attenuated by Fer-1 or FPS-ZM1 treatment (Figure 2C). Furthermore, Liperfluo staining indicated that CA04 infection enhanced lipid peroxidation-related oxidative signals and reduced the intensity of reductive signals, whereas both Fer-1 and FPS-ZM1 significantly decreased the oxidative signal compared with the CA04 group (Figure 2D).

### 3.3. RAGE Inhibitor Mitigates Influenza Virus-Induced Pro-Inflammatory Polarization by Suppressing Ferroptosis

**Figure 3:**
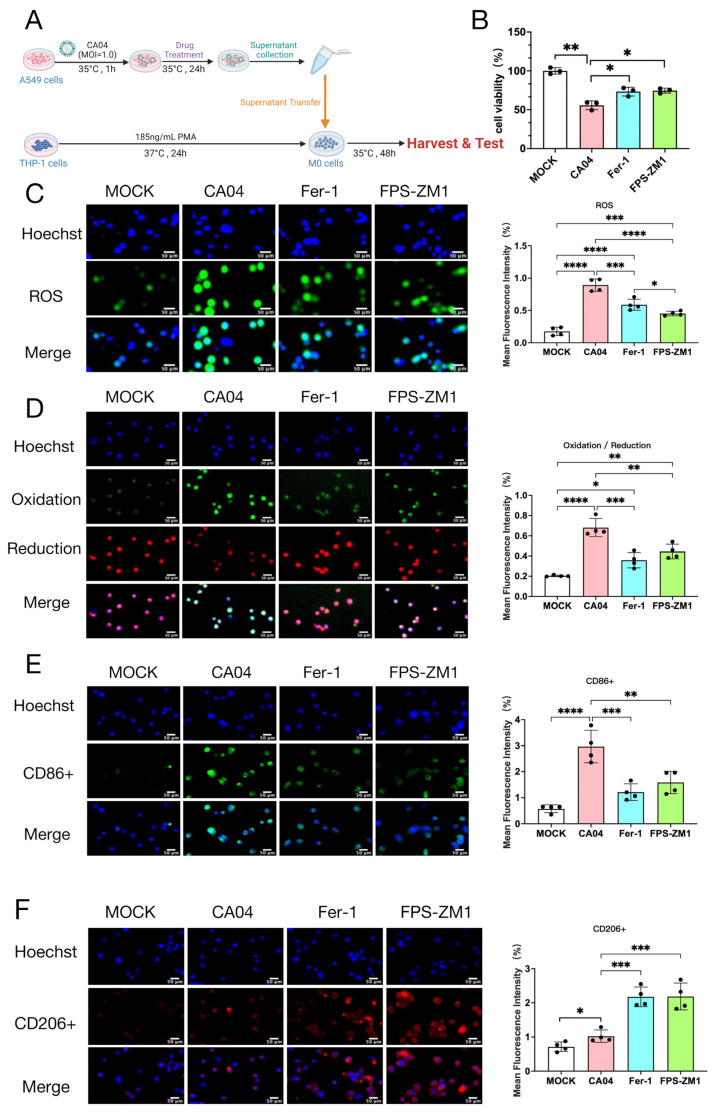
Effects of FPS-ZM1 and Fer-1 on THP-1–derived M0 macrophages treated with CA04-infected A549 supernatant. (**A**) Experimental design schematic. (**B**) Cell viability of THP-1–derived M0 macrophages treated with different concentrations of FPS-ZM1 (*n* = 4). (**C**) Immunofluorescence detection of intracellular ROS (green) and nuclei (DAPI, blue) in THP-1–derived M0 macrophages treated with supernatant from CA04-infected A549 cells following 24 h treatment with Fer-1 or FPS-ZM1 (*n* = 4). Scale bar = 50 μm. (**D**) Immunofluorescence detection of lipid peroxidation in THP-1–derived M0 macrophages treated with supernatant from CA04-infected A549 cells following 24 h treatment with Fer-1 or FPS-ZM1. Representative images show oxidized lipids (green), reduced lipids (red), and nuclei (DAPI, blue). Quantification represents the ratio of oxidized to reduced BODIPY 581/591 C11 fluorescence (*n* = 4). Scale bar = 50 μm. (**E**,**F**) Immunofluorescence detection of pro-inflammatory M1 marker CD86 (**E**) and anti-inflammatory M2 marker CD206 (**F**) in macrophages treated with CA04-infected A549 cell supernatant following 24 h treatment with Fer-1 or FPS-ZM1 (*n* = 4). Scale bar = 50 μm. Data are presented as mean ± standard deviation (SD). Statistical comparisons were performed between each treatment group and the CA04-infected group. * *p* < 0.05, ** *p* < 0.01, *** *p* < 0.001, **** *p* < 0.0001.

To investigate the impact of epithelial RAGE activation on macrophage responses, A549 cells were infected as indicated, and the conditioned media were subsequently collected and applied to PMA-differentiated THP-1–derived M0 macrophages (Figure 3A). Cell viability assays showed that M0 cells treated with supernatants from CA04-infected A549 cells exhibited a significant decrease in viability, whereas Fer-1 or FPS-ZM1 treatment significantly improved cell survival compared with the CA04 group (Figure 3B). ROS measurement showed that CA04 supernatant treatment markedly increased ROS accumulation in M0 cells, which was significantly attenuated by Fer-1 or FPS-ZM1 (Figure 3C). Liperfluo staining indicated enhanced lipid peroxidation in CA04-treated M0 cells, while the oxidative signal was substantially reduced in Fer-1- and FPS-ZM1-treated groups (Figure 3D).

Previous studies have suggested that ferroptosis promotes M1 pro-inflammatory polarization of macrophages [23,24]. To further examine the effect of RAGE inhibition on macrophage polarization, the proportions of M1-type (CD86^+^) and M2-type (CD206^+^) macrophages were analyzed in THP-1–derived macrophages exposed to conditioned media. The results showed that supernatants from CA04-infected A549 cells increased both M1 and M2 proportions in M0 macrophages, suggesting an overall activation state with a dominant pro-inflammatory bias. In contrast, Fer-1 or FPS-ZM1 treatment significantly decreased the M1 fraction while increasing the M2 fraction (Figure 3E,F).

### 3.4. RAGE Inhibitor Reduces Ferroptosis in PR8-Infected Mice

**Figure 4:**
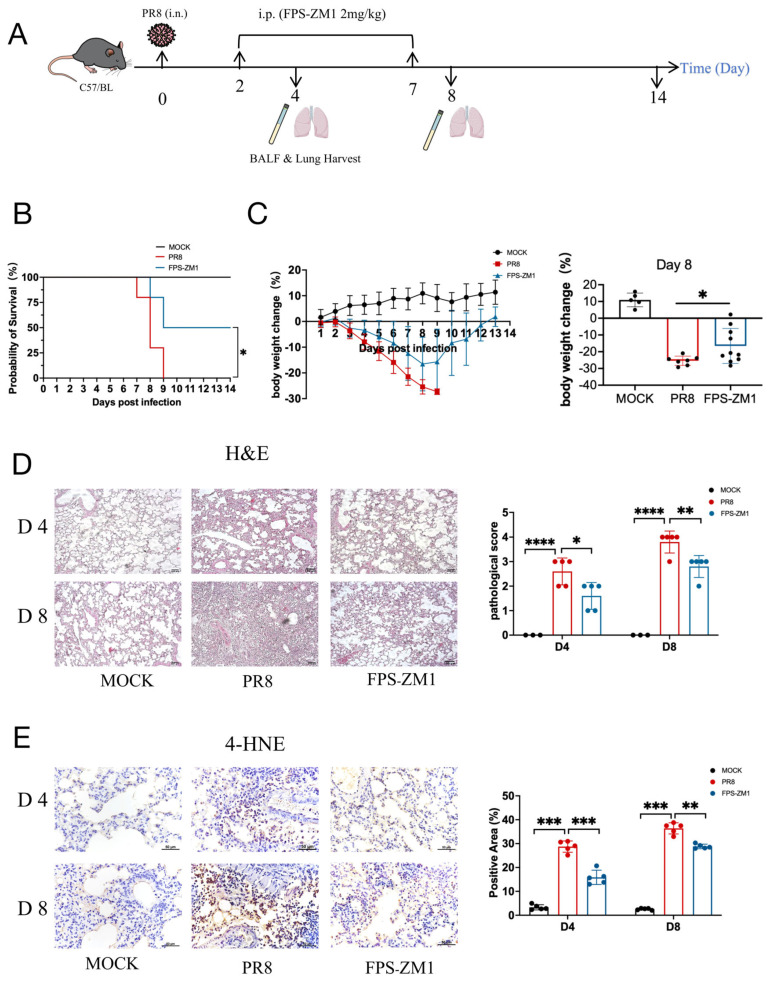

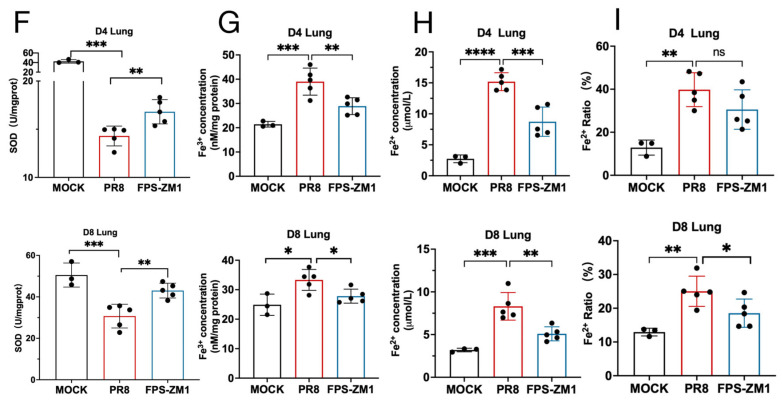
FPS-ZM1 alleviates lung injury and ferroptosis in mice following PR8 infection. (**A**) Schematic illustration of the experimental design. Mice were challenged with the PR8 strain via intranasal (i.n.) administration, and FPS-ZM1 or vehicle was administered via intraperitoneal (i.p.) injection. (**B**,**C**) Survival rate ((**B**), *n* = 10) and body weight change ((**C**), *n* = 10) of PR8-infected mice. (**D**) Representative H&E staining of lung tissues showing histopathological changes (*n* = 5) Scale bar = 100 μm. (**E**) Immunohistochemical detection of 4-HNE in lung tissues (*n* = 5). Scale bar = 50 μm. (**F**,**I**) Measurements of SOD activity (**F**), Fe^3+^ (**G**), Fe^2+^ (**H**), and Fe^2+^/Fe^3+^ ratio (**I**) in lung tissues (*n* = 5). Data are presented as mean ± standard deviation (SD). Statistical analyses were performed using the PR8 group as control. * *p* < 0.05, ** *p* < 0.01, *** *p* < 0.001, **** *p* < 0.0001.

Our previous study demonstrated that the RAGE inhibitor FPS-ZM1 decreases both the pathogenicity and lethality of mice following PR8 infection [18]. In this study, we treated PR8-infected mice with a higher dose of FPS-ZM1 (2 mg/kg vs. 1 mg/kg in the previous protocol) daily for five consecutive days starting from day 2 post-infection (Figure 4A). The results showed that high-dose FPS-ZM1 exhibited superior efficacy compared with the previous regimen. Survival analysis showed that all mice in the vehicle-treated group (PR8 group) succumbed to infection by day 9 post-infection, whereas the FPS-ZM1-treated group exhibited a 50% survival rate at day 14, representing a 30% improvement over the low-dose regimen (Figure 4B). Body weight monitoring revealed a continuous decline in the PR8 group, reaching the lowest point on day 8 post-infection. In contrast, mice treated with FPS-ZM1 exhibited a remarkably smaller loss of body weight (Figure 4C). Histopathological analysis of lung tissues showed pronounced structural damage and extensive inflammatory cell infiltration in the PR8 group on days 4 and 8 post-infection, whereas lung architecture in the FPS-ZM1 group remained relatively preserved, with markedly less inflammatory cell infiltration (Figure 4D). These findings suggest that the higher FPS-ZM1 dosage confers enhanced protection.

Immunohistochemical staining showed that 4-hydroxynonenal (4-HNE), a major lipid peroxidation product and biomarker of ferroptosis [25], was significantly elevated in the PR8 group, whereas FPS-ZM1 treatment markedly reduced 4-HNE expression in lung tissues of mice on days 4 and 8 post-infection (Figure 4E). Furthermore, superoxide dismutase (SOD), a key antioxidant enzyme that protects against oxidative stress [26], was significantly decreased in the PR8 group, whereas FPS-ZM1 treatment partially restored SOD activity (Figure 4F). Iron measurements revealed significant increases in both Fe^2+^ and Fe^3+^ in the PR8 group, which were reduced by FPS-ZM1 treatment (Figure 4G,H). Since Fe^2+^ is the catalytically active form of iron that drives lipid peroxidation through Fenton chemistry [2,27], we further analyzed the Fe^2+^/Fe^3+^ ratio. Analysis showed no significant difference between the PR8 and FPS-ZM1 groups on day 4 post-infection; however, a significantly lower Fe^2+^/Fe^3+^ ratio was observed in FPS-ZM1-treated mice on day 8 post-infection (Figure 4I).

### 3.5. RAGE Inhibitor Modulates Expression of Key Ferroptosis Regulators ACSL4, POR, and GPX4

**Figure 5:**
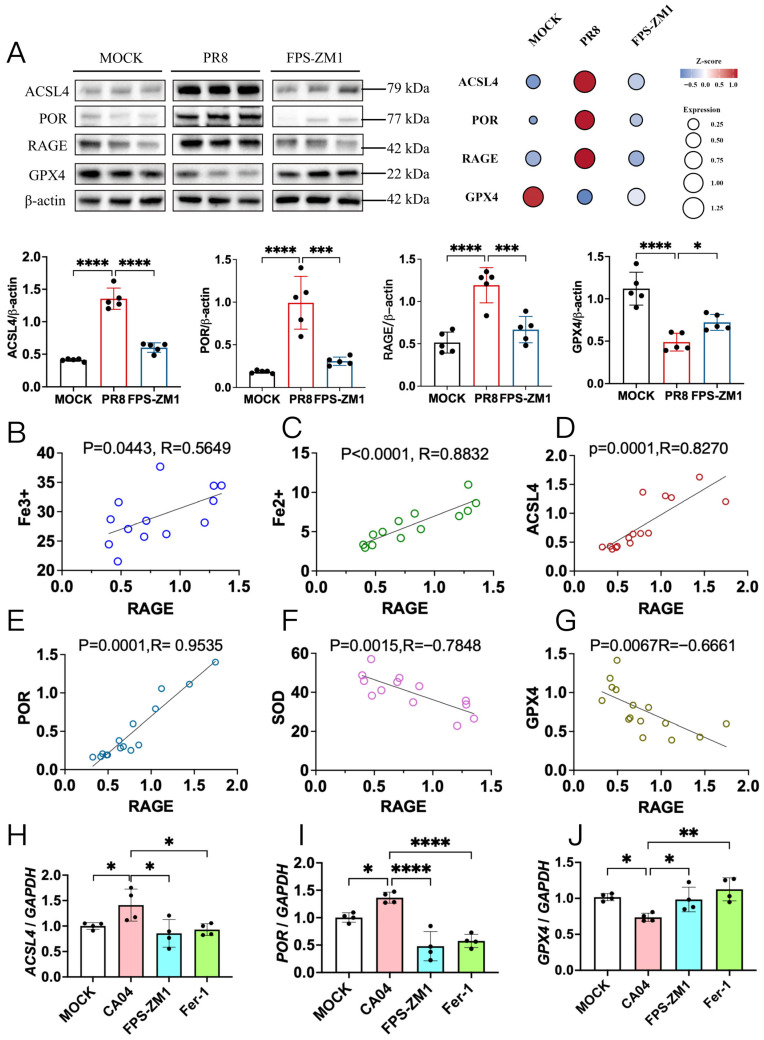

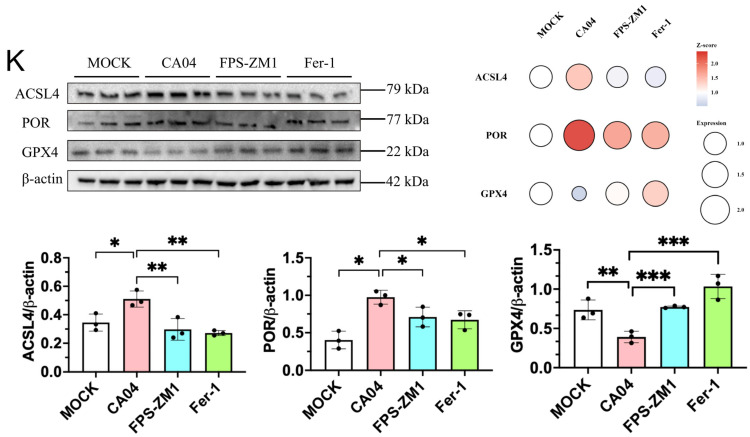
FPS-ZM1 reduces ferroptosis following H1N1 infection via the ACSL4/POR/GPX4 pathway. (**A**) Western blot analysis of key ferroptosis proteins ACSL4, POR, RAGE and GPX4 in lung tissues of mice on day 8 post-PR8 infection (Representative blots of *n* = 3 are shown). (**B**–**G**) Correlation analysis between lung RAGE levels and Fe^3+^ (**B**), Fe^2+^ (**C**), ACSL4 (**D**), POR (**E**), SOD (**F**), and GPX4 (**G**) on day 8 post-PR8 infection (*n* = 15). (**H**–**J**) qRT-PCR analysis of *ACSL4* (**H**), *POR* (**I**), and *GPX4* (**J**) in CA04-infected A549 cells 24 h after treatment with FPS-ZM1 or Fer-1 (*n* = 4). (**K**) Western blot analysis of ACSL4, POR, and GPX4 in CA04-infected A549 cells 24 h after treatment with FPS-ZM1 or Fer-1 (*n* = 3). Data are presented as mean ± standard deviation (SD). Statistical comparisons were performed between the vehicle-treated PR8-infected group and the FPS-ZM1-treated group in vivo, and between the CA04-infected group and treatment groups in vitro * *p* < 0.05, ** *p* < 0.01, *** *p* < 0.001, **** *p* < 0.0001.

To further investigate the molecular mechanisms by which RAGE regulates ferroptosis, we examined the expression of key ferroptosis-related proteins in the lungs of mice 8 days post-infection. Acyl-CoA synthetase long-chain family member 4 (ACSL4) and cytochrome P450 oxidoreductase (POR) drive lipid peroxidation and ferroptotic cell death by promoting polyunsaturated fatty acid (PUFA) activation and H_2_O_2_ generation, respectively [28,29]. In contrast, glutathione peroxidase 4 (GPX4) serves as a critical host protective factor in ferroptosis by reducing lipid peroxidation and preventing cell death [30]. Western blot analysis revealed that ACSL4, POR, and RAGE were significantly upregulated in the lungs of PR8-infected mice, whereas GPX4 levels were markedly reduced. Treatment with the RAGE inhibitor FPS-ZM1 significantly increased GPX4 expression while decreasing ACSL4, POR and RAGE levels (Figure 5A). Correlation analysis demonstrated that RAGE expression was positively associated with Fe^3+^, Fe^2+^, ACSL4, and POR in lung tissues (Figure 5B–E), while negatively correlated with SOD activity and GPX4 expression (Figure 5F,G).

Consistently, in CA04-infected A549 cells, qRT-PCR analysis showed that both *ACSL4* and *POR* mRNA levels were significantly increased in the CA04 group, and were markedly reduced by FPS-ZM1 or Fer-1 treatment (Figure 5H,I). Conversely, *GPX4* mRNA levels were significantly decreased in the untreated CA04 group compared with the MOCK group, whereas FPS-ZM1 or Fer-1 treatment restored *GPX4* expression (Figure 5J). Western blot results corroborated these findings at the protein level (Figure 5K).

### 3.6. RAGE Inhibitor Decreases the Accumulation of Pro-Inflammatory M1 Macrophages and Neutrophils in the Lungs of PR8-Infected Mice

**Figure 6:**
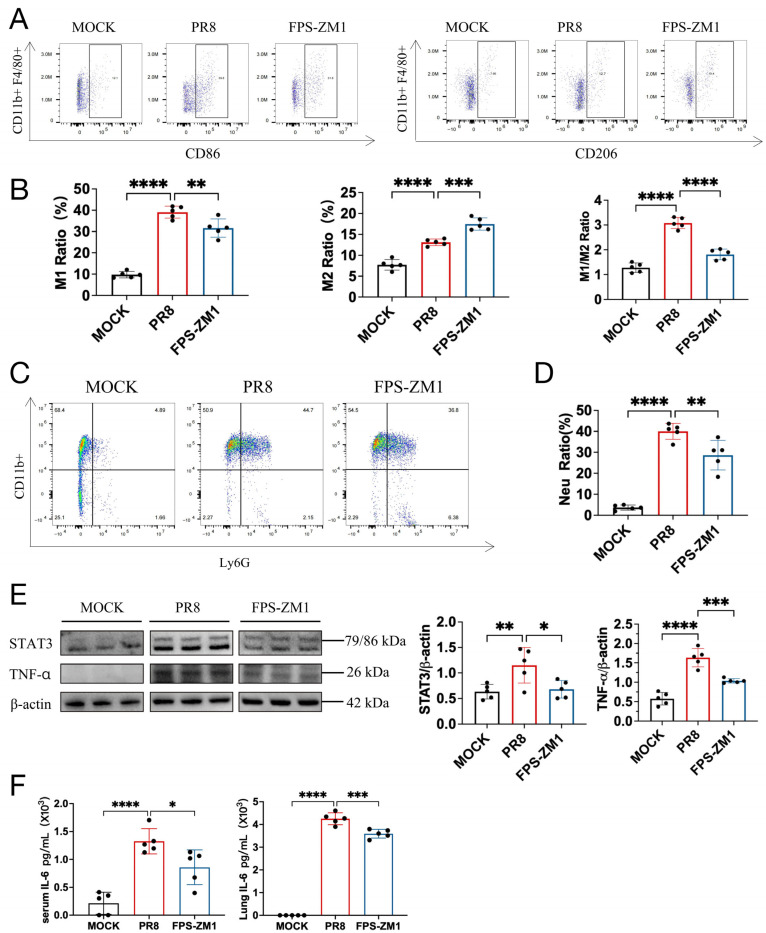
FPS-ZM1 alters macrophage polarization in the lungs of PR8-infected mice. (**A**) Flow cytometric analysis of M1 (CD45^+^CD11b^+^F4/80^+^CD86^+^) and M2 (CD45^+^CD11b^+^F4/80^+^CD206^+^) macrophages in bronchoalveolar lavage fluid (BALF) from mice on day 8 post-PR8 infection (*n* = 5). (**B**) Proportion of M1, M2, and M1/M2 ratio in BALF from mice on day 8 post-PR8 infection (*n* = 5). (**C**) Flow cytometric analysis of neutrophils (CD45^+^CD11b^+^Ly6G^+^) in BALF on day 8 post-PR8 infection (*n* = 5). (**D**) The proportion of neutrophils in BALF on day 8 post-PR8 infection (*n* = 5). (**E**) Western blot analysis of TNF-α and STAT3 Protein levels in lung tissues on day 8 post-PR8 infection (*n* = 5). (**F**) IL-6 levels in serum and lung tissues on day 8 post-PR8 infection as determined by ELISA. (*n* = 5). Data are presented as mean ± standard deviation (SD). Statistical analyses were performed using the PR8 group as control, * *p* < 0.05, ** *p* < 0.01, *** *p* < 0.001, **** *p* < 0.0001.

Given that in vitro treatment with the RAGE inhibitor reduced the proportion of M1-polarized macrophages while increasing M2 polarization (Figure 3E,F), we further assessed alveolar macrophages in BALF from mice using flow cytometry. Similarly, PR8 infection significantly increased the proportion of M1-polarized macrophages in BALF on day 8 post-infection. In contrast, FPS-ZM1 treatment significantly decreased the proportion of M1 macrophages (CD86^+^) while increasing the proportion of M2 macrophages (CD206^+^) (Figure 6A,B). Accordingly, the M1/M2 ratio in FPS-ZM1-treated mice was much lower than that in the PR8 group (Figure 6B).

Given the critical role of RAGE in innate immunity, we also evaluated myeloid-derived neutrophils in BALF. Neutrophil numbers were significantly increased following PR8 infection, whereas FPS-ZM1 treatment significantly reduced neutrophil levels (Figure 6C,D).

Analysis of M1-associated effector molecules showed that lung expression of STAT3 and TNF-α was markedly increased in the PR8 group, but significantly decreased in FPS-ZM1-treated mice, consistent with the hub genes identified in the RAGE–ferroptosis interaction network analysis (Figure 1B and Figure 6E). Additionally, IL-6 levels in both serum and lung tissue were significantly elevated in the PR8 group on day 8 post-infection, whereas FPS-ZM1 treatment significantly reduced IL-6 levels (Figure 6F).

## 4. Discussion

While RAGE is well-established as a pro-inflammatory mediator in acute lung injury, its mechanistic link to ferroptosis has remained unexplored. The present findings reveal functional crosstalk between RAGE signaling and ferroptotic cell death during IAV infection, expanding our understanding of RAGE beyond classical inflammation. This connection is particularly noteworthy given that both RAGE activation and ferroptosis have been independently implicated in ARDS and severe viral pneumonia, suggesting convergent pathogenic mechanisms that may be therapeutically targetable.

RAGE inhibition attenuates virus–associated lung injury, potentially through modulation of ferroptosis. In vitro experiments demonstrated that pharmacological blockade of RAGE improved cell survival and reduced ferroptosis-associated oxidative stress, with effects comparable to the canonical ferroptosis inhibitor Ferrostatin-1 (Fer-1), suggesting a close mechanistic linkage between RAGE signaling and ferroptotic activation. In macrophages, RAGE inhibition suppressed pro-inflammatory M1 polarization, consistent with previous reports demonstrating ferroptosis-related inflammatory regulation across multiple cell types [31]. In vivo, RAGE inhibition significantly alleviated lung injury and reduced lipid peroxidation, supporting a contributory role of RAGE in ferroptosis-driven tissue damage. These findings are consistent with previous studies, which have reported that ginkgolide B alleviates sepsis-induced ferroptosis and acute lung injury by targeting RAGE [32], while astragalside II pretreatment has also been shown to ameliorate PM2.5-induced lung injury through suppression of ferroptosis in mice [33]. Collectively, these results support a model in which RAGE-associated ferroptosis may contribute to virus-induced lung injury through enhancement of oxidative stress [15,33,34,35,36].

Molecular association analysis indicated that RAGE may influence ferroptosis through regulation of the ACSL4/POR/GPX4 axis. ACSL4 and POR are recognized drivers of lipid peroxidation, whereas GPX4 serves as a central anti-ferroptotic regulator that detoxifies phospholipid hydroperoxides [36,37,38]. Previous studies have demonstrated that upregulation of ACSL4, downregulation of GPX4, and accumulation of lipid peroxides constitute hallmark molecular features of ferroptosis in inflammatory models such as acute lung injury (ALI) [36,39,40], with POR further recognized as an indispensable mediator and a potential therapeutic target in ferroptotic cell death [41]. In the present study, RAGE inhibition consistently suppressed ACSL4 and POR expression while restoring GPX4 levels in both infected lungs and epithelial cells, supporting a regulatory association between RAGE signaling and ferroptotic activation. Importantly, our data suggest a temporally modulated pattern in which RAGE signaling influences iron homeostasis during the course of infection. Although the magnitude of change was modest, statistically significant differences in the Fe^2+^/Fe^3+^ ratio between early and later time points suggest a progressive shift toward catalytically active Fe^2+^, which could contribute to enhanced Fe^2+^-dependent lipid peroxidation at later stages. While early ferroptotic responses may be primarily driven by acute oxidative stress, later phases appear to involve stage-related modulation of iron redox status and ferroptotic processes. These observations are consistent with previous reports demonstrating that advanced glycation end products (AGE)/RAGE signaling promotes inflammation-associated ferroptosis and that RAGE blockade restores GPX4 expression and alleviates ferroptotic injury [32].

Oxidative stress and inflammatory responses represent key mechanistic interfaces linking RAGE activation to ferroptosis. Bioinformatic analyses revealed significant enrichment of overlapping RAGE–ferroptosis genes within ROS metabolism and inflammation-related pathways, with TP53, EGFR, BCL2, JUN, STAT3, and IL6 emerging as potential hub mediators. These findings suggest that RAGE activation may coordinate ferroptotic signaling and immune responses through redox-dependent networks. Unlike apoptosis and necrosis, ferroptosis is triggered when intracellular ROS levels reach a lethal threshold [37], and excessive ROS has been shown to promote macrophage polarization toward a pro-inflammatory M1 phenotype under diverse inflammatory conditions, such as intracellular or extracellular pH alterations and activation of TLR2/4 signaling [42,43,44]. In the present study, RAGE inhibition attenuated ROS accumulation and reduced M1 polarization in epithelial and myeloid cells, supporting a functional link between redox imbalance and immune skewing. Moreover, ROS accumulation has been reported to enhance transcription of proinflammatory cytokines, including IL-6 and TNF-α, through activation of the IL-6/STAT3 signaling pathway, thereby reinforcing M1 polarization in diverse inflammatory contexts [45,46,47]. Consistent with this framework, elevated STAT3 and IL-6 expression in infected lungs was suppressed by RAGE inhibition, aligning with previous reports that RAGE activation enhances IL-6/STAT3 signaling in lung injury models [48]. Taken together, our data support a model in which a RAGE-associated ROS–ferroptosis–macrophage polarization signaling network amplifies inflammatory responses during influenza virus infection and may represent a key regulatory component underlying acute lung injury.

Beyond influenza, these findings suggest broader translational implications. Oxidative stress and dysregulated inflammation are shared pathological features of multiple acute respiratory infections, including those caused by IAV and SARS-CoV-2 [49,50,51]. RAGE is highly expressed in lung tissue under pathological conditions and functions as a pattern-recognition receptor involved in multiple signaling pathways, including inflammatory activation, apoptosis, autophagy, and ferroptosis [18,48]. Aberrant RAGE activation promotes ROS accumulation and excessive inflammatory responses, thereby exacerbating pulmonary injury [52]. Similar mechanisms have also been reported in models of chronic inflammatory disorders. For example, sustained RAGE signaling contributes to macrophage M1 polarization and oxidative stress in diabetic atherosclerosis models, and RAGE blockade attenuates this pathological process [53]. Importantly, compared with our previous low-dose regimen (1 mg/kg) [18], the optimized higher-dose FPS-ZM1 treatment (2 mg/kg) resulted in improved survival and reduced pathology. This dose-dependent efficacy is consistent with previous studies demonstrating that RAGE antagonist therapeutic outcomes are influenced by dosing strategies in inflammatory disease models [54,55]. Optimization of RAGE-targeted strategies could therefore enhance protection against severe inflammatory lung injury. Collectively, these observations support the potential of RAGE modulation as a host-directed approach in respiratory diseases characterized by excessive oxidative stress and immune imbalance [56].

Nonetheless, this study has several limitations. First, the causal relationship between RAGE and key ferroptosis regulators remains to be directly validated, and direct genetic evidence for RAGE-mediated regulation of ferroptosis is still limited. Future studies incorporating genetic manipulation approaches will be required to establish definitive causal links. Second, the upstream signaling pathways and precise molecular mechanisms underlying RAGE–ferroptosis crosstalk have not been fully elucidated. Future studies aimed at dissecting these signaling networks will provide deeper mechanistic insight into RAGE-dependent regulation of ferroptosis during influenza infection.

In summary, our findings suggest that RAGE contributes to ferroptosis-associated oxidative stress and immune dysregulation during IAV-induced lung injury. Modulation of the RAGE–ferroptosis axis may therefore represent a potential host-directed strategy for limiting inflammation-driven tissue damage.

## 5. Conclusions

This study demonstrates that RAGE activation drives ferroptosis-mediated lung injury during influenza virus infection through the ACSL4/POR/GPX4 axis, establishing a self-reinforcing pathological circuit in which ferroptosis and inflammatory responses mutually amplify one another through macrophage polarization and pro-inflammatory cytokine production. Pharmacological inhibition of RAGE using FPS-ZM1 effectively disrupts this vicious cycle, attenuating ferroptotic cell death, restoring redox homeostasis, and rebalancing macrophage polarization in both in vitro and in vivo models. Collectively, these findings identify the RAGE–ferroptosis axis as a critical regulatory node in virus-induced acute lung injury and establish RAGE as a promising therapeutic target with broad translational potential in severe pulmonary infections characterized by excessive inflammation and oxidative damage.

## Figures and Tables

**Table 1:** Details of the top 15 results of KEGG.

ID	Description	Count	q Value
hsa05131	Shigellosis	11	1.03 × 10^−7^
hsa01522	Endocrine resistance	8	1.05 × 10^−7^
hsa05417	Lipid and atherosclerosis	10	1.21 × 10^−7^
hsa04066	HIF-1 signaling pathway	8	1.22 × 10^−7^
hsa05212	Pancreatic cancer	7	1.95 × 10^−7^
hsa04068	FoxO signaling pathway	8	3.61 × 10^−7^
hsa05215	Prostate cancer	7	7.54 × 10^−7^
hsa04933	AGE-RAGE signaling pathway in diabetic complications	7	8.15 × 10^−7^
hsa04218	Cellular senescence	8	8.79 × 10^−7^
hsa05206	MicroRNAs in cancer	10	1.32 × 10^−6^
hsa01521	EGFR tyrosine kinase inhibitor resistance	6	3.32 × 10^−6^
hsa05169	Epstein–Barr virus infection	8	4.63 × 10^−6^
hsa05210	Colorectal cancer	6	4.63 × 10^−6^
hsa05235	PD-L1 expression and PD-1 checkpoint pathway in cancer	6	5.26 × 10^−6^
hsa05224	Breast cancer	7	5.91 × 10^−6^

**Table 2:** Details of the top GO enrichment.

Ontology	ID	Description	Count	q Value
BP	GO:2000628	regulation of miRNA metabolic process	9	1.42 × 10^−10^
BP	GO:2000377	regulation of reactive oxygen species metabolic process	10	1.48 × 10^−10^
BP	GO:0036294	cellular response to decreased oxygen levels	10	1.48 × 10^−10^
BP	GO:0010586	miRNA metabolic process	9	1.48 × 10^−10^
BP	GO:0072593	reactive oxygen species metabolic process	11	1.65 × 10^−10^
CC	GO:0030139	endocytic vesicle	9	1.85 × 10^−6^
CC	GO:0017053	transcription repressor complex	5	2.10 × 10^−5^
CC	GO:0005741	mitochondrial outer membrane	6	1.35 × 10^−4^
CC	GO:0019867	outer membrane	6	1.42 × 10^−4^
CC	GO:0030666	endocytic vesicle membrane	5	6.73 × 10^−4^
MF	GO:0001228	DNA-binding transcription activator activity, RNA polymerase II-specific	9	3.26 × 10^−5^
MF	GO:0031625	ubiquitin protein ligase binding	7	1.10 × 10^−4^
MF	GO:0140297	DNA-binding transcription factor binding	8	1.53 × 10^−4^
MF	GO:0019903	protein phosphatase binding	5	2.66 × 10^−4^
MF	GO:0001227	DNA-binding transcription repressor activity, RNA polymerase II-specific	6	3.39 × 10^−4^

BP: biological process, CC: cellular component, MF: molecular function.

## Data Availability

The data presented in this study are openly available in GeneCards (https://www.genecards.org/ (accessed on 29 March 2026)) and STRING 11.0 at https://string-db.org/ (accessed on 29 March 2026). The raw data supporting the conclusions of this article are available from the corresponding author on request.

## Supplementary Materials

The following supporting information can be downloaded at: https://www.mdpi.com/article/10.3390/antiox15040434/s1, Figure S1: Cytotoxicity assessment of FPS-ZM1 and Fer-1 in A549 and THP-1 cells; Figure S2: Original uncropped Western blot membranes; Figure S3: Gating strategy for flow cytometry.
