# Supplementary material for: The RAGE–Ferroptosis Axis Drives Oxidative Stress-Associated Inflammatory Lung Injury in Viral Infection
Source: Antioxidants (Basel). 2026 Mar 31;15(4):434. doi: 10.3390/antiox15040434 (PMC13114047; doi:10.3390/antiox15040434)
Supplement: Supplementary file 1 [file antioxidants-15-00434-s001.zip › antioxidants-4145381-supplementary.pdf]

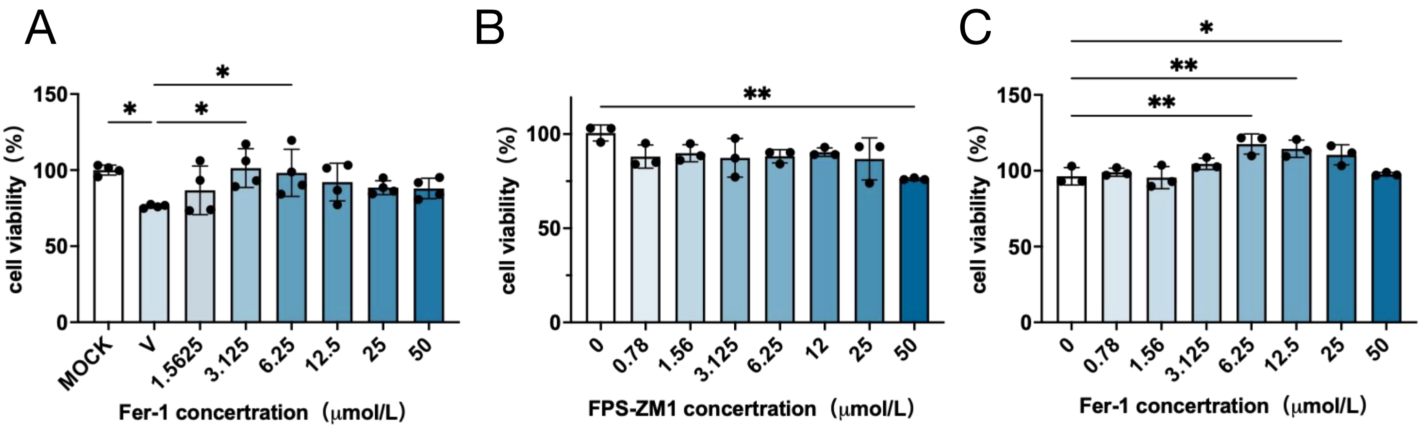

Figure S1. Cytotoxicity assessment of FPS-ZM1 and Fer-1 in A549 and THP-1 cells. (A) Cell viability of CA04-infected *A549* cells treated with different concentrations of Fer-1 (n = 4). (B) Cell viability of *THP-1* cells treated with different concentrations of FPS-ZM1 (n = 3). (C) Cell viability of *THP-1* cells treated with different concentrations of Fer-1 (n = 3). Data are presented as mean ± SD. For statistical analysis, the control group was the virus (V) group for *A549* cells and the MOCK group for *THP-1* cells. *p < 0.05, **p < 0.01.

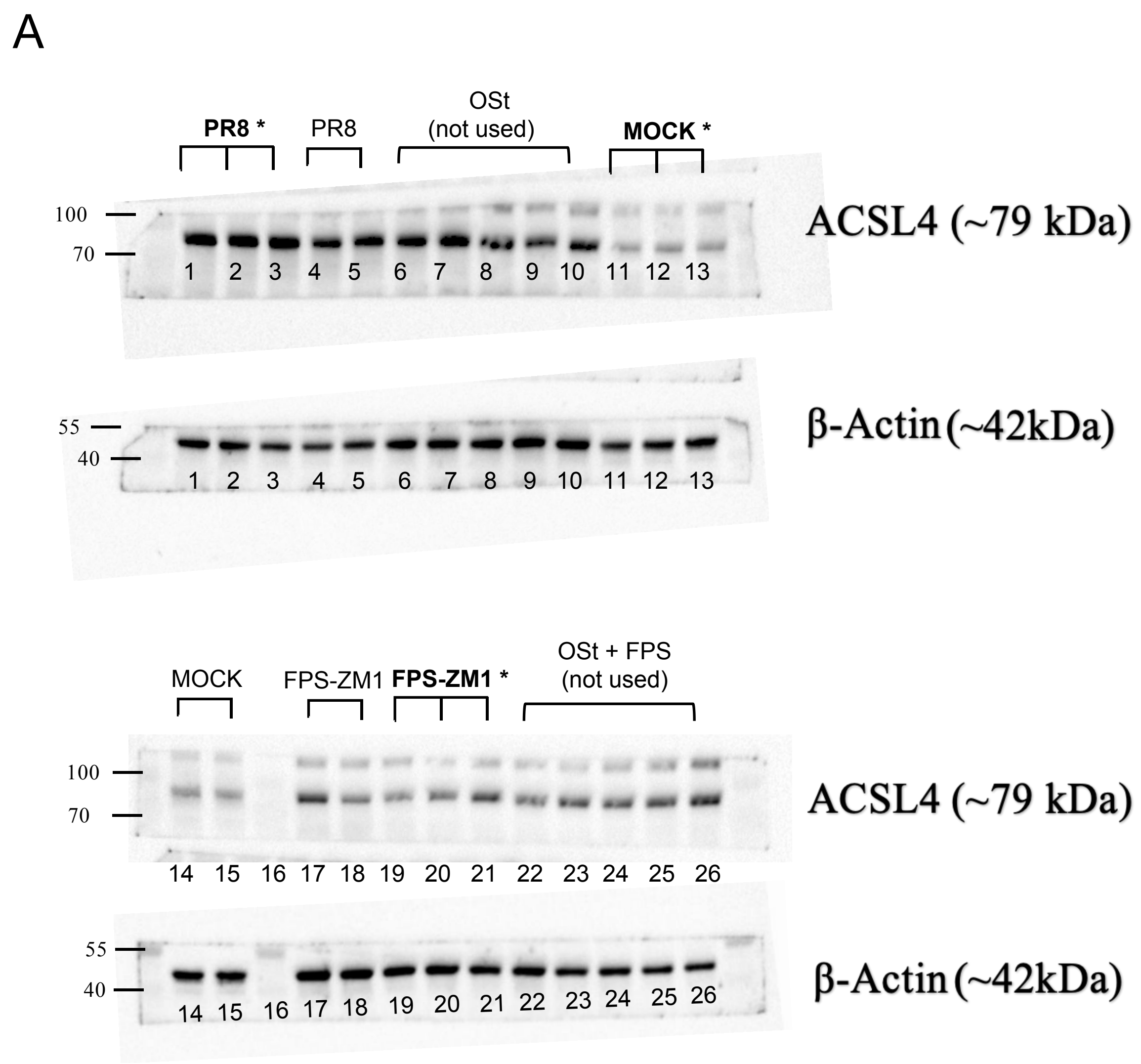

* lanes shown in the main figures

MW marker: Thermo Scientific PageRuler Prestained Protein Ladder (10–180 kDa, Cat. No. 26616)

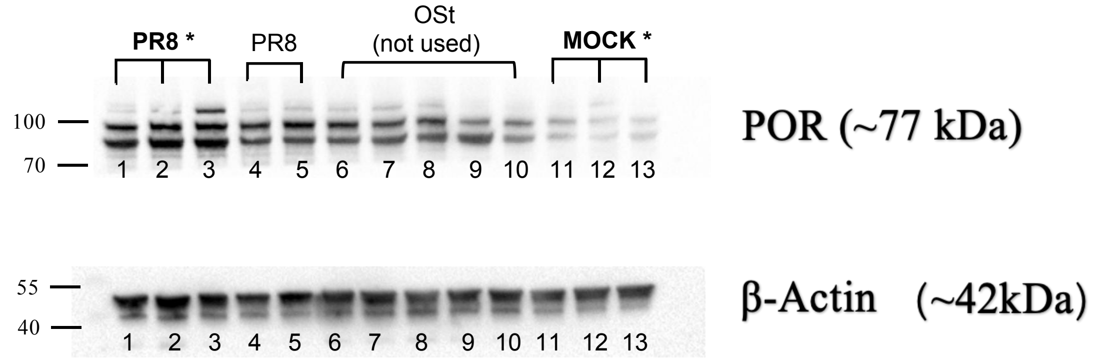

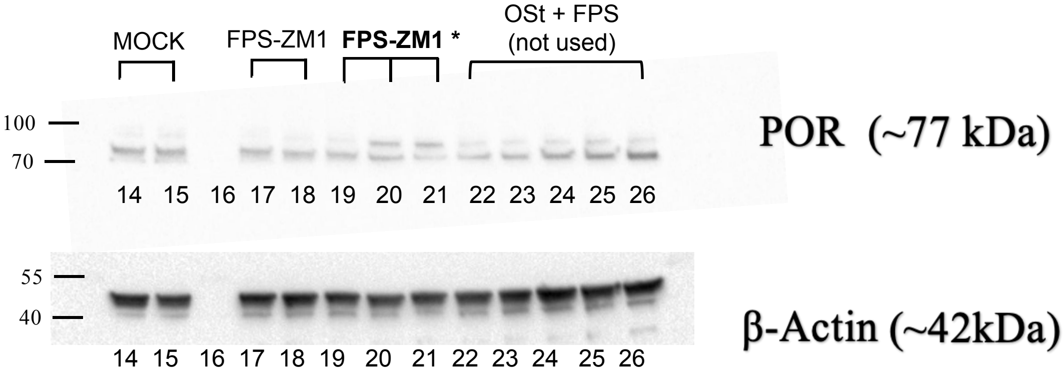

* lanes shown in the main figures

MW marker: Thermo Scientific PageRuler Prestained Protein Ladder (10–180 kDa, Cat. No. 26616)

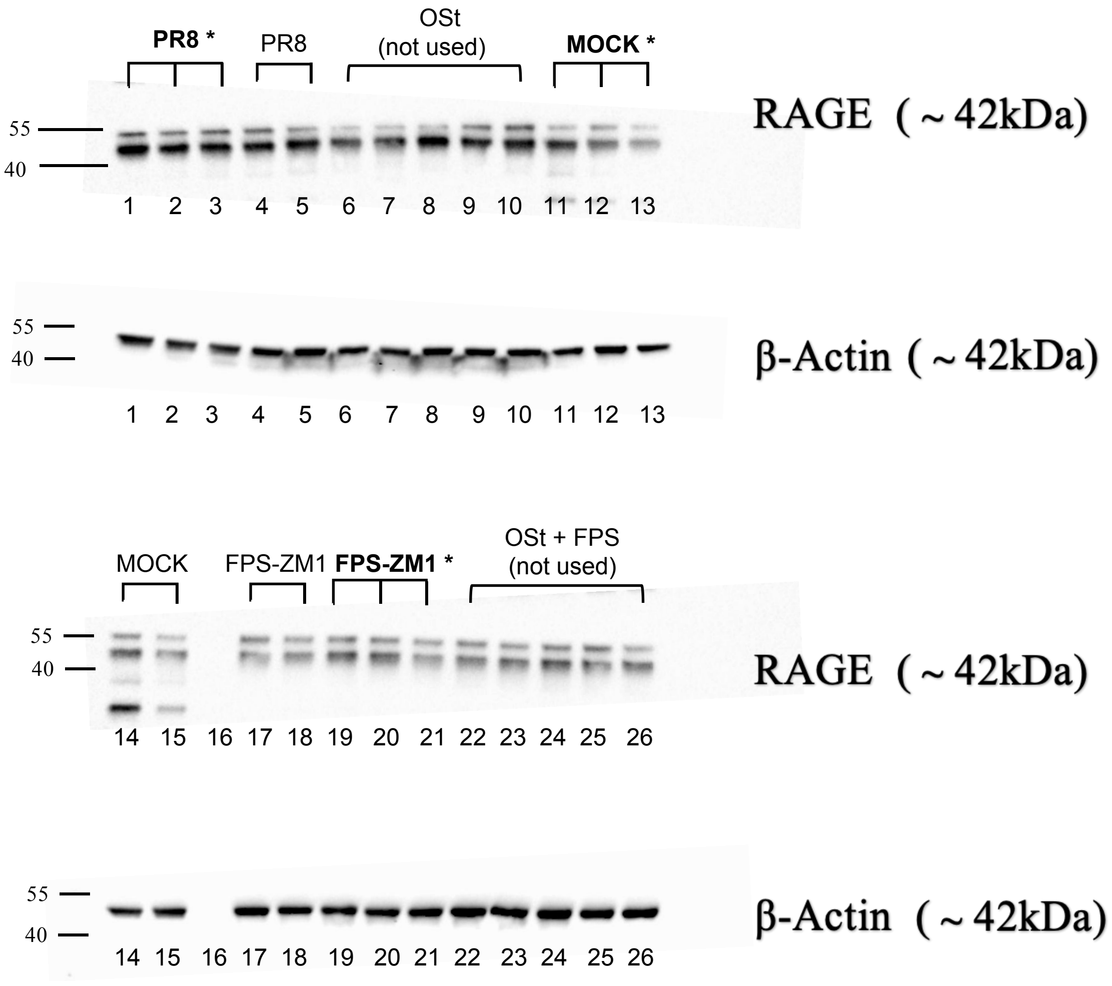

■ The RAGE blot and β-actin loading control were obtained from **different gels but from the same experimental run under identical conditions.**

* lanes shown in the main figures

MW marker: Thermo Scientific PageRuler Prestained Protein Ladder (10–180 kDa, Cat. No. 26616)

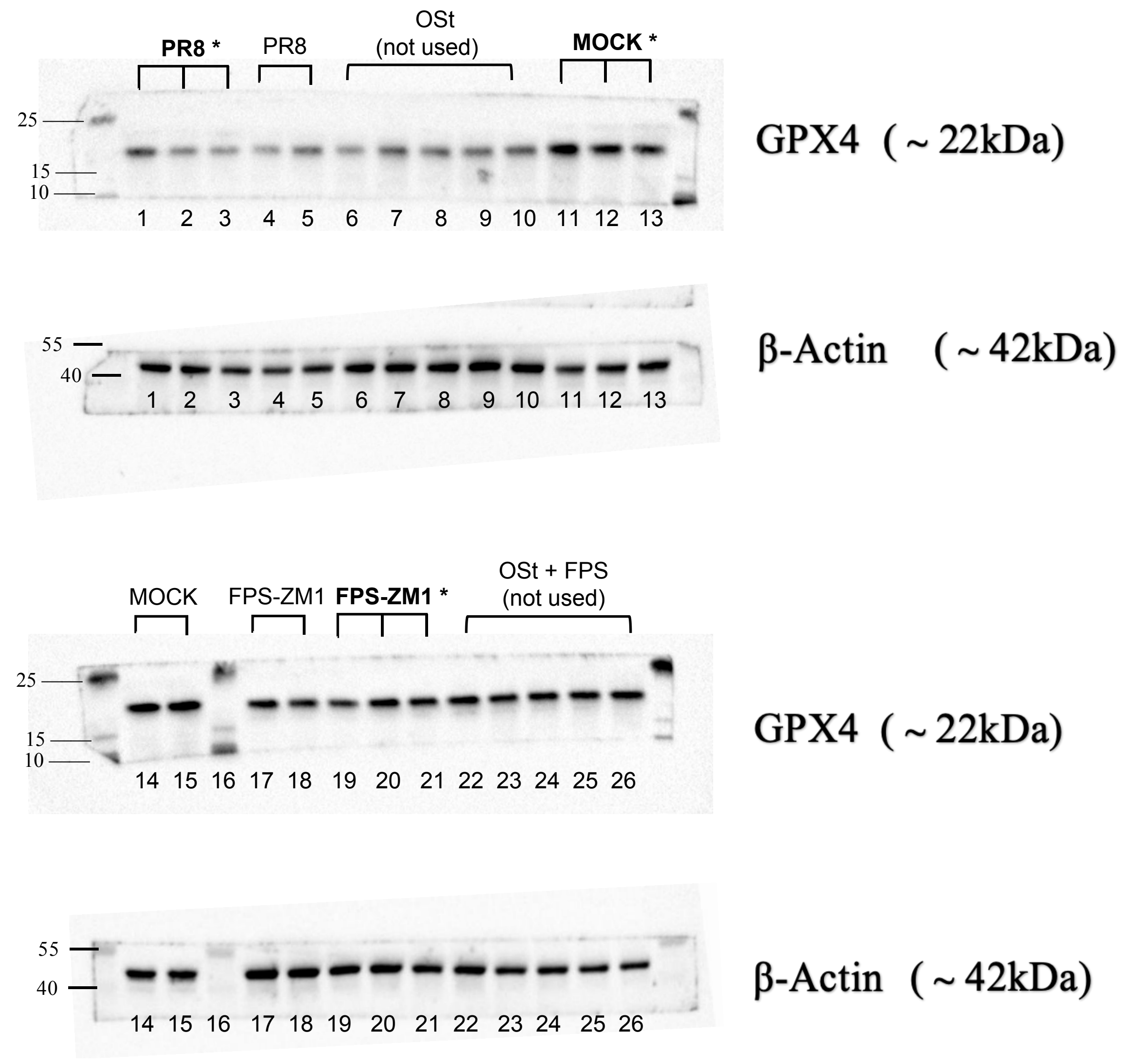

* lanes shown in the main figures

MW marker: Thermo Scientific PageRuler Prestained Protein Ladder (10–180 kDa, Cat. No. 26616)

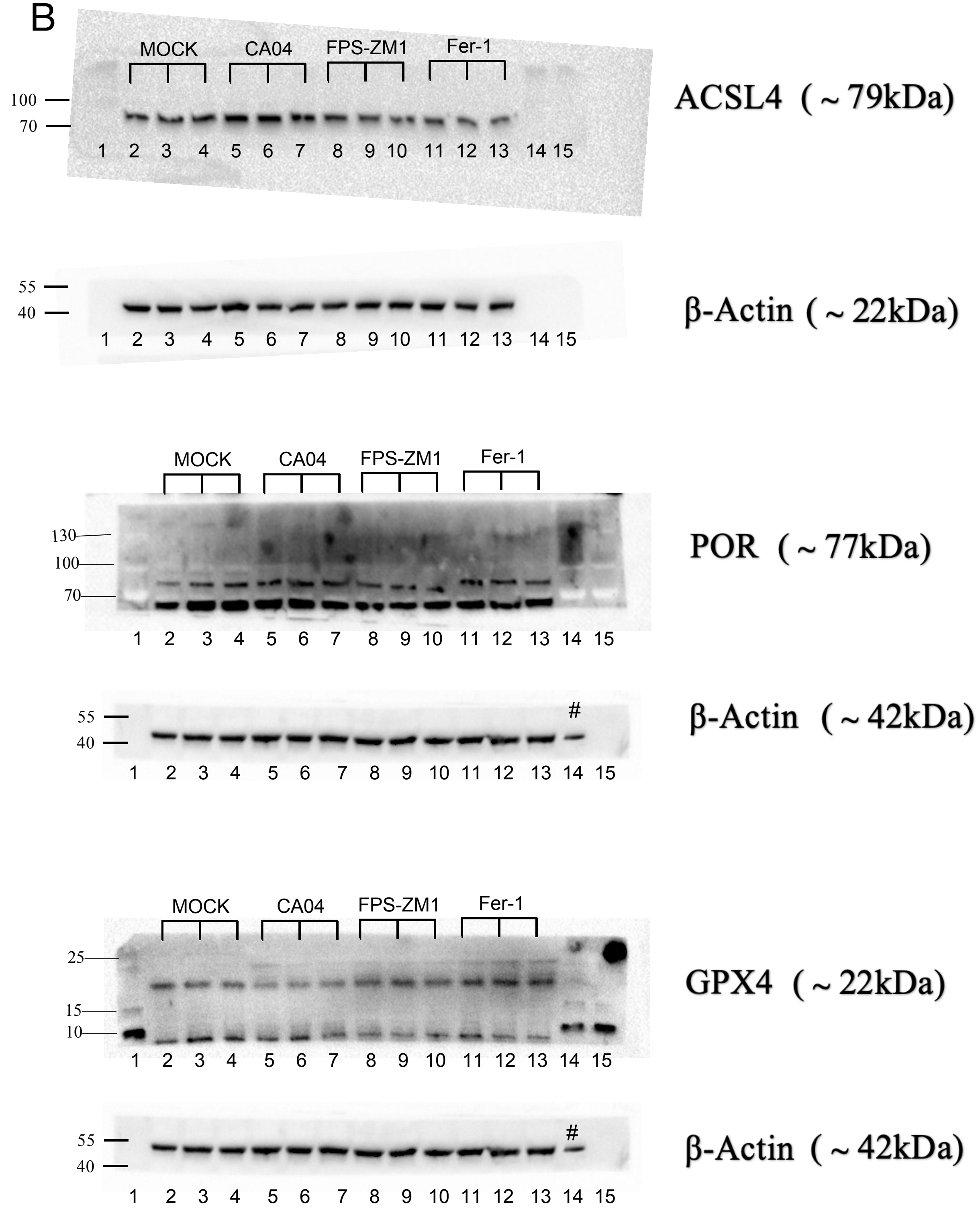

* lanes shown in the main figures

MW marker: Thermo Scientific PageRuler Prestained Protein Ladder (10–180 kDa, Cat. No. 26616)

Additional signal in lane 14 attributed to slight sample diffusion from the adjacent lane during loading.

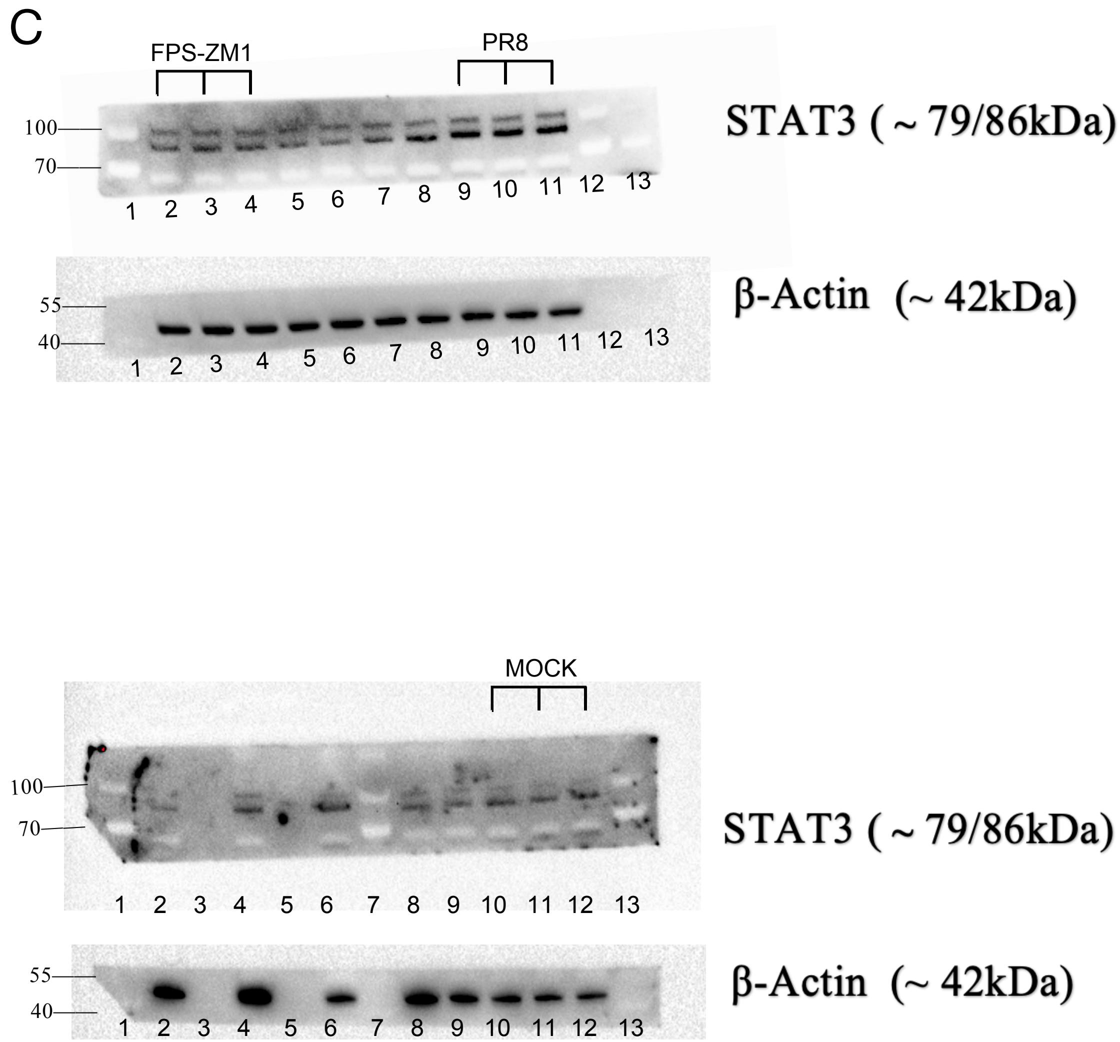

* lanes shown in the main figures

MW marker: Thermo Scientific PageRuler Prestained Protein Ladder (10–180 kDa, Cat. No. 26616)

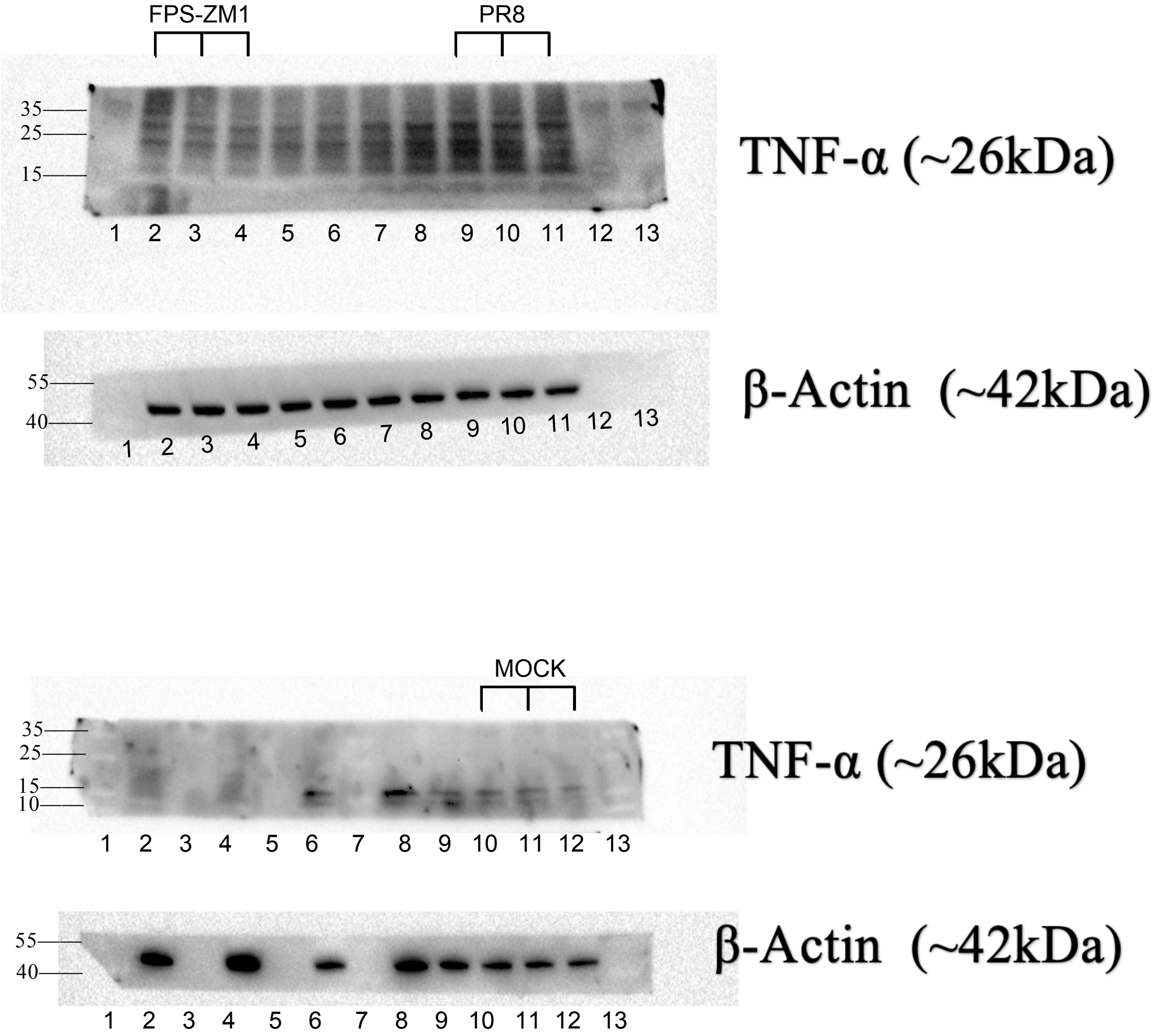

Figure S2. Original uncropped Western blot membranes. (A) Western blot analysis of ACSL4, POR, RAGE, and GPX4 in whole-lung lysates from PR8-infected mice treated with FPS-ZM1 (*n* = 5). (A) Western blot analysis of ACSL4, POR, and GPX4 in whole-cell lysates from CA04-infected *A549* cells treated with FPS-ZM1 or Fer-1 for 24 h (*n* = 3). (C) Western blot analysis of STAT3 and TNF-α in whole-lung lysates from PR8-infected mice treated with FPS-ZM1 (*n* = 5). β-Actin was used as the loading control for all panels.

* lanes shown in the main figures

MW marker: Thermo Scientific PageRuler Prestained Protein Ladder (10–180 kDa, Cat. No. 26616)

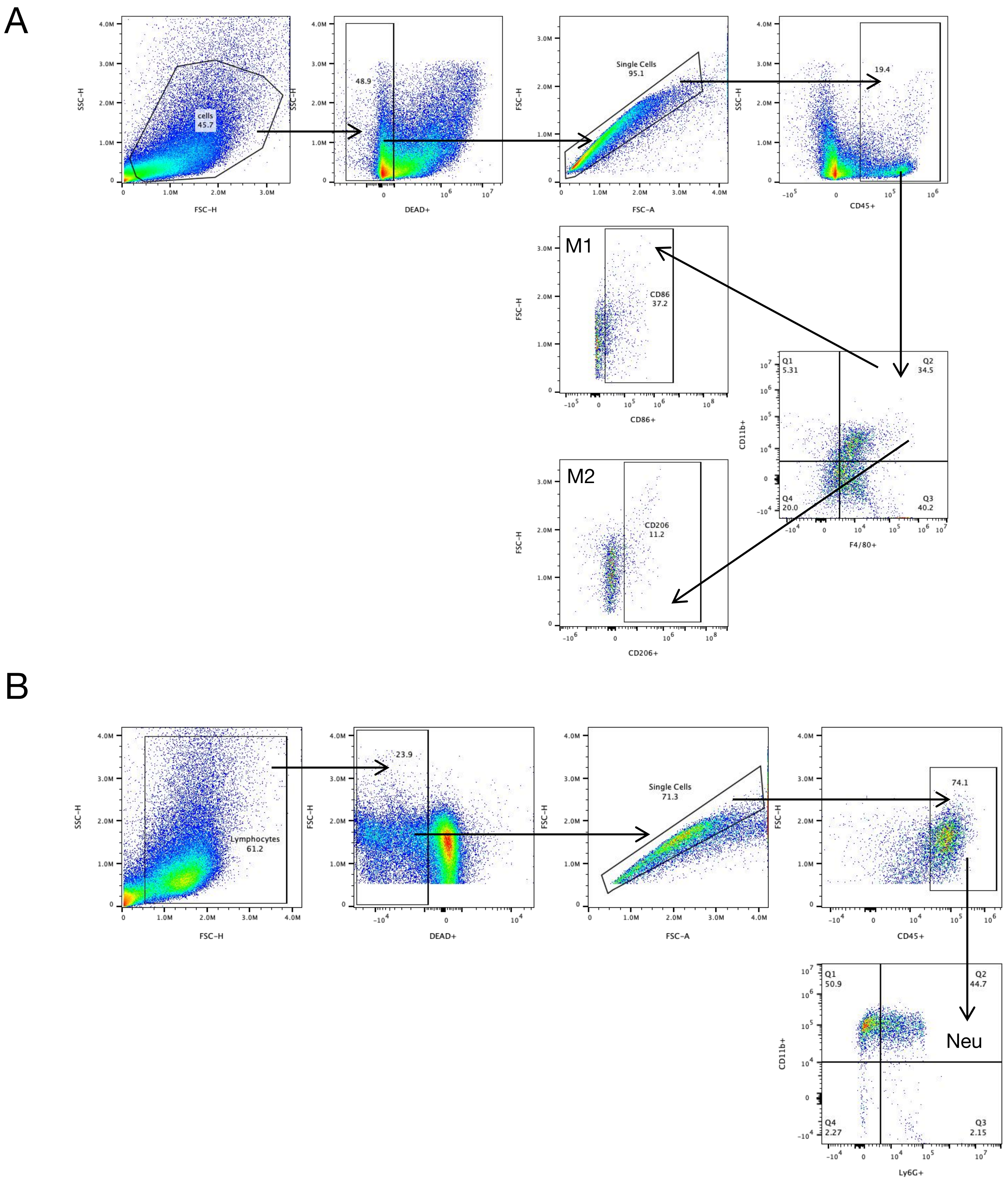

Figure S3. Gating strategy for flow cytometry. (A) Gating strategy for identification of macrophage populations (M1: $CD45^{+}CD11b^{+}F4/80^{+}CD86^{+}$; M2: $CD45^{+}CD11b^{+}F4/80^{+}CD206^{+}$) in bronchoalveolar lavage fluid (BALF). (B) Gating strategy for identification of neutrophils ($CD45^{+}CD11b^{+}Ly6G^{+}$) in BALF.
